# Advanced Corneal Hydrogels: From Passive Replacement to Active Regeneration and Intelligent Interaction

**DOI:** 10.1002/advs.202522362

**Published:** 2026-02-25

**Authors:** Xinwei Wang, Wen Zhou, Haotian Xue, Wenjun Xue, Guangping Gao, Bing Zhu, Hongyuan Song, Qingqiang Xu, Jing Zhao, Wei Shen

**Affiliations:** ^1^ Department of Ophthalmology, Shanghai Changhai Hospital The First Affiliated Hospital of Naval Medical University Shanghai P. R. China; ^2^ School of Medicine Tongji University Shanghai P. R. China; ^3^ Department of Plastic Surgery, Shanghai Changhai Hospital The First Affiliated Hospital of Naval Medical University Shanghai P. R. China; ^4^ Lab of Toxicology and Pharmacology, Faculty of Naval Medicine Naval Medical University Shanghai P. R. China; ^5^ Eye Institute and Department of Ophthalmology Eye & ENT Hospital Fudan University Shanghai P. R. China; ^6^ Key Laboratory of Myopia and Related Eye Diseases NHC Shanghai China; ^7^ Key Laboratory of Myopia and Related Eye Diseases Chinese Academy of Medical Sciences Shanghai P. R. China

**Keywords:** 3D printing, artificial cornea, biomaterial, cornea, drug delivery, Hydrogel, tissue engineering

## Abstract

Hydrogels represent a transformative solution for corneal pathologies, offering the advantages of transparency, biocompatibility, and structural tunability to address the global donor tissue shortage. This review establishes a comprehensive framework for material design by delineating critical requirements, including optical clarity, mechanical anisotropy, and stable wet adhesion within dynamic ocular environments. The performances of natural, synthetic, and decellularized‐matrix hydrogels are systematically compared to elucidate the structure–property relationships essential for recapitulating native corneal physiology. Beyond passive substitution, the transition toward bioactive regeneration is examined through analyses of scarless wound healing, sustained drug delivery, and stimuli‐responsive implants. Furthermore, the integration of advanced technologies is evaluated with a focus on three‐/four‐dimensional bioprinting for hierarchical architectural reconstruction and hydrogel‐based wearable bioelectronics for real‐time sensing. Finally, pivotal clinical translation bottlenecks, ranging from sterilization to long‐term immunocompatibility, are identified to guide the development of next‐generation personalized and intelligent corneal devices.

## Graphical abstract

This review charts the evolution of corneal hydrogels from passive replacements to active and intelligent systems, evaluating natural and synthetic materials while addressing design challenges such as transparency and strength. Advanced applications such as scarless wound healing, three‐dimensional bioprinting, and smart wearables are highlighted to guide the creation of future personalized ocular therapies.

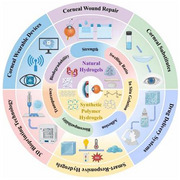

## 1 Introduction

The cornea is a transparent, avascular, and immune‐privileged tissue with rich sensory innervation, accounting for approximately two‐thirds of the eye's total refractive power [1]. Its topographic curvature, optical transparency, and structural regularity are critical determinants of visual acuity [2]. Pathologies and structural abnormalities in the cornea can cause various vision impairments, including refractive errors [3], infectious keratitis [4, 5], traumatic corneal injuries [6], dry eye–associated keratopathy, keratoconus, and corneal dystrophies. At least 2.2 billion people around the world experience vision impairment, which results in an estimated annual productivity loss of up to USD 411 billion [7]. At least 1 billion of these cases could have been prevented or remain unaddressed [8]. Corneal blindness is the third global leading cause of blindness after cataract and glaucoma, with ∼10 million people affected bilaterally [9].

Corneal diseases are primarily managed with pharmacological (mainly topical eye drops) and surgical interventions. The residence time of eye drops on the ocular surface is limited by blinking and tear clearance—a drawback that cannot be fully mitigated by increasing the administration frequency or formulation concentration. Corneal transplantation remains the primary surgical treatment after the irreversible loss of corneal transparency [7]. However, only 1 in 70 patients receives the needed graft [9]. Penetrating keratoplasty has been increasingly superseded by lamellar keratoplasty, which selectively replaces the diseased layers of the cornea [7]. However, the supply from eye banks remains severely limited, especially in developing countries [7].

**FIGURE 1:**
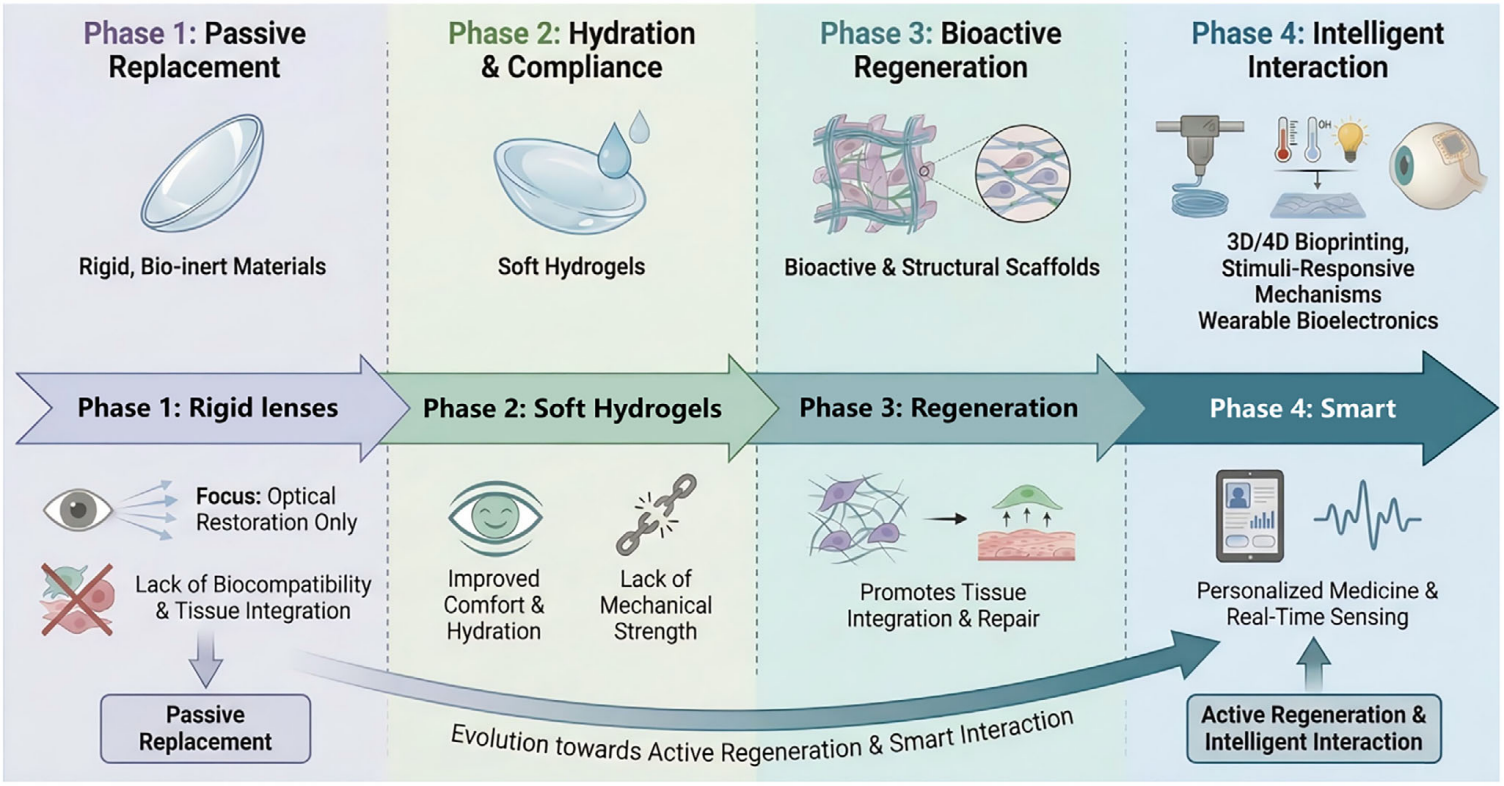
Evolutionary trajectory of hydrogel materials for corneal applications illustrating a transition from passive replacement to active regeneration and intelligent interaction.

Hydrogels are three‐dimensional (3D) network‐structured materials with a high water content but solid‐like properties. Despite the notable potential of hydrogels, their widespread clinical use in corneal repair is restricted by several critical bottlenecks. One of the primary challenges is the material property trilemma: High mechanical strength is often achieved at the expense of optical transparency and biocompatibility, which hinders the full replication of the anisotropic and robust nature of native cornea [10, 11]. Furthermore, the ocular surface presents a unique, dynamic, and wet microenvironment where strong long‐term interfacial adhesion is difficult to achieve without suturing [12, 13]. Current solutions struggle to perfectly synchronize hydrogel degradation rates with the physiological corneal healing cycle, which results in premature scaffold failure or impeded tissue regeneration [14]. Addressing these limitations requires a shift from passive material substitution to active functional reconstruction (Figure 1).

**FIGURE 2:**
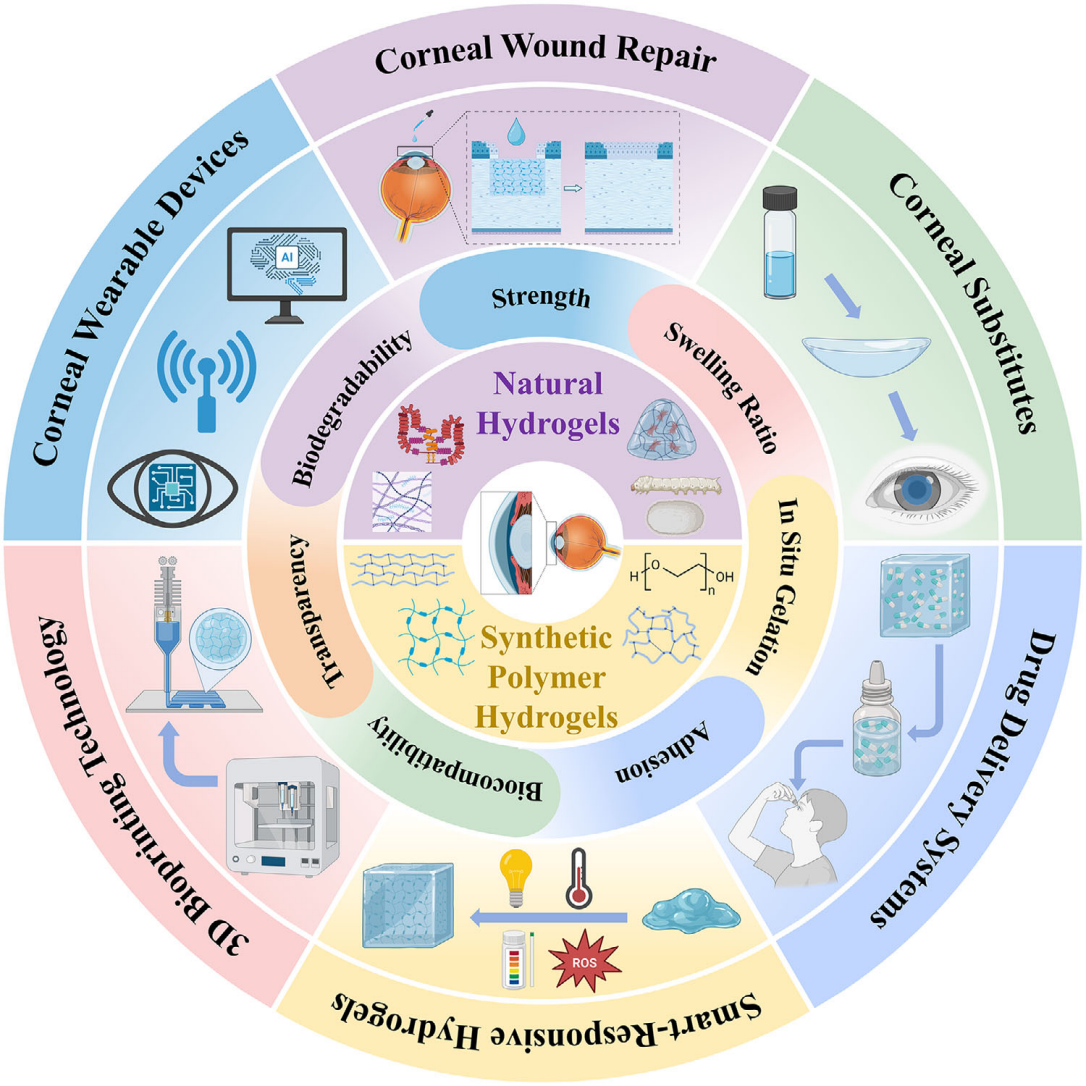
Corneal hydrogels: Properties, material classification, and applications (created with BioRender.com).

To capture the evolution of this field and address the abovementioned challenges, we present a comprehensive analysis of corneal hydrogels supported by a conceptual timeline illustrating their evolution from passive replacement to intelligent functionalized systems. This review systematically categorizes hydrogels according to material source and elucidates the structure–property relationships essential for overcoming current performance barriers (Figure 2). We critically examine cutting‐edge applications, including scarless wound healing, functional corneal substitutes, intelligent drug delivery platforms, and stimuli‐responsive implants, and highlight the transformative potential of integration with 3D bioprinting and wearable technologies. Finally, we analyze the technical hurdles hindering clinical adoption and propose future research directions, thus providing a perspective on designing the next generation of personalized intelligent corneal devices.

## 2 Corneal Anatomy and Repair Mechanisms

### 2.1 Corneal Structure and Function

**FIGURE 3:**
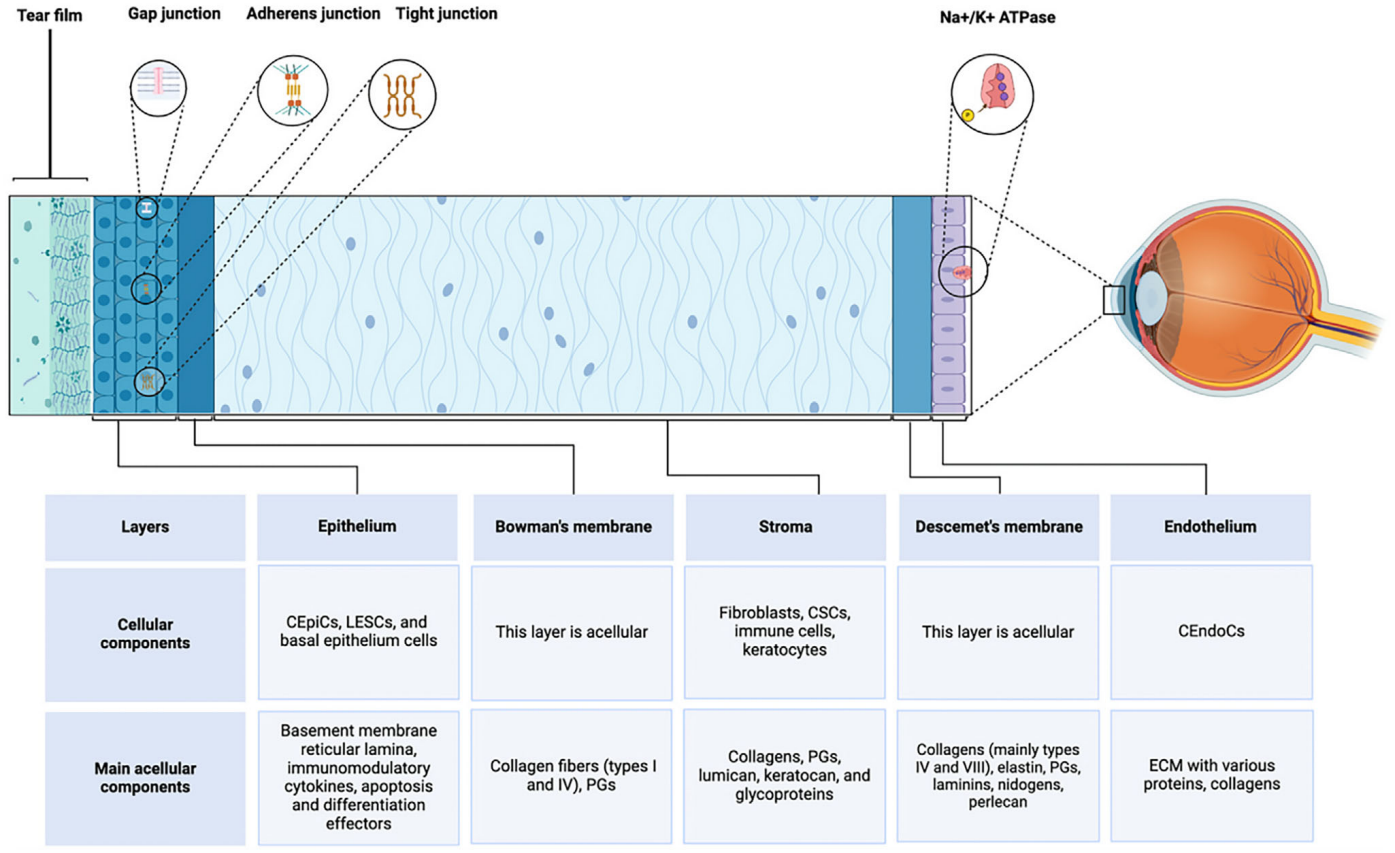
Structure and composition of the human cornea. Adapted with permission [1]. Copyright 2025, Elsevier.

The cornea, which constitutes approximately one‐sixth of the fibrous outer layer of the eye, is normally thinnest at the center and thickest at the periphery (average thickness ≈ 0.5 and 1 mm, respectively) and becomes thinner with age. This tissue is important for maintaining ocular integrity, protecting intraocular contents, transmitting and refracting light, and sensing environmental and external stimuli [2, 15, 16] and comprises five layers (anterior to posterior: corneal epithelium, Bowman's layer, stroma, Descemet's membrane, and corneal endothelium) (Figure 3). The corneal epithelium is a nonkeratinized, nonsecretory, stratified squamous epithelium typically comprising 5–7 layers [15] and is further divided into the cellular layer and epithelial basement membrane (EBM). The cellular layer comprises three sublayers (inner to outer: basal cells, wing cells, and superficial squamous cells). Corneal epithelial cells serve as a barrier and provide support, stabilizing the tear film. It also protects internal ocular structures from microorganisms, blunt trauma, and toxic chemicals [15, 16]. Bowman's layer is a relatively homogeneous acellular collagenous fibrous membrane located beneath the EBM. Composed of collagen fibrils and proteoglycans, this layer exhibits strong resistance to mechanical force but is vulnerable to chemical damage and cannot be regenerated after injury [15]. The stroma is the most highly organized and transparent tissue in the human body, featuring a thickness of ∼500 µm and accounting for ∼90% of the corneal thickness. This tissue comprises collagen fibrils, keratocytes, mucoproteins, and glycoproteins [17]. The precise arrangement of the collagen fibrils is essential for corneal transparency. Descemet's membrane, situated behind the stroma and extending peripherally to Schwalbe's line at the anterior chamber angle, is secreted by corneal endothelial cells (CEndoCs) and has regenerative capacity after injury [18]. In contrast to Bowman's layer, Descemet's membrane is less resistant to mechanical trauma but more resistant to chemical and pathological insults. The weak adhesion of this membrane to the stroma and corneal endothelium renders it susceptible to detachment in cases of trauma or certain pathological conditions. The corneal endothelium—the innermost layer of the cornea—comprises a monolayer of hexagonal cuboidal epithelial cells interconnected by tight junctions and functions as a barrier, preventing aqueous humor from penetrating the extracellular space while actively pumping out fluid to maintain corneal deturgescence and transparency. Corneal endothelial cells also lack regenerative capacity; repair occurs only through cell migration and expansion. Considerable cell loss may lead to decompensation and thus cause corneal edema and bullous keratopathy [15, 18].

### 2.2 Pathophysiology of Corneal Injury and Repair Mechanisms

**FIGURE 4:**
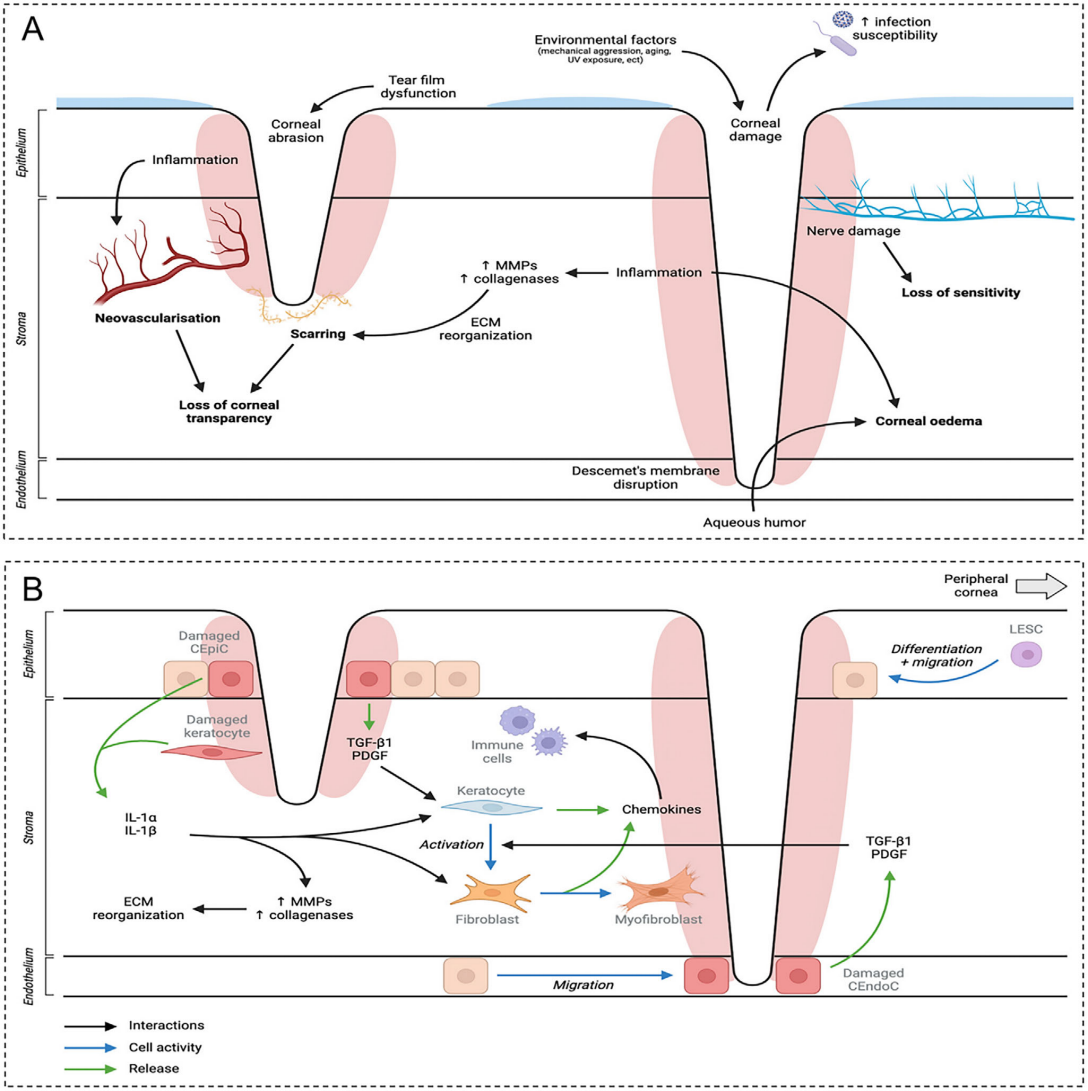
Schematic of corneal injury repair. (A) Causes and biological consequences of corneal damage. Adapted with permission [1]. Copyright 2025, Elsevier. (B) Physiological healing process of the cornea. Adapted with permission [1]. Copyright 2025, Elsevier.

Corneal diseases primarily include inflammation, infection, trauma, congenital anomalies, degeneration, dystrophy, and neoplasms (Figure 4A). The limbal region is highly vascularized and features higher concentrations of lymphocytes and complement components than the central cornea. The corneoscleral limbus harbors antigen‐presenting dendritic cells, while vascular adhesion molecules and cytokines recruit various leukocytes from circulation to the limbus. Consequently, immune‐mediated corneal diseases predominantly occur in the peripheral or limbus, whereas infectious keratopathies are more likely to affect the central cornea. The continuous renewal of the corneal epithelium relies on limbal stem cells, which undergo clonal proliferation to generate centripetally migrating cell populations, thereby maintaining homeostasis in the central corneal region [16, 19]. Following injury to the corneal epithelium, limbal stem cells differentiate into transient amplifying cells and accelerate cell migration to the wound site via the activation of the P38 and ERK1/2 signaling pathways [20]. The PI3K‐AKT signaling pathway suppresses scar formation mediated by TGF‐β and PDGF‐β [21], while ferroptosis inhibition mitigates oxidative stress–induced epithelial damage, promoting morphological and functional recovery [22]. The integrity of the corneal EBM is critical for preventing fibrosis. Bioactive hydrogels can mimic the extracellular matrix (ECM) microenvironment and facilitate well‐organized epithelial regeneration [23]. Additionally, chitosan membranes markedly enhance corneal transparency and thickness restoration by providing physical support and bioactive signaling [24]. As mentioned earlier, Bowman's layer does not regenerate after injury and is instead replaced by epithelial cells or scar tissue. Following damage to the corneal stroma, corneal stromal cells and fibroblasts mediate the rearrangement of collagen fibers through molecules such as MYLK, MYL9, and ITGA3, forming an orthogonal structure to restore transparency. Additionally, these cells regulate growth factor (e.g., TGF‐β and PDGF‐β) signaling pathways to reduce the production of scar‐type ECM, inhibiting stromal fibrosis and scar formation [24] (Figure 4B). Certain synthetic hydrogels can mimic the physical properties of the native corneal stroma, supporting the regeneration of epithelial and stromal layers while suppressing fibrosis [25]. Corneal stromal stem cells or fibroblasts can be induced to differentiate into functional corneal stromal cells through serum factors or fibrous hydrogels, thereby promoting stromal regeneration [26]. Descemet's membrane can be regenerated after injury through secretion by endothelial cells. In adults, corneal endothelial cells possess negligible proliferative capacity; hence, damaged areas are primarily repaired through cell migration, morphological expansion, and functional compensation. This inherent limitation prevents complete regeneration following severe injury, often necessitating exogenous interventions such as transplantation or cell injection [15, 27]. Rho‐associated protein kinase (ROCK) inhibitors can reduce apoptosis and enhance the adhesion and spread of corneal endothelial cells by suppressing the ROCK signaling pathway [28]. Hydrogel biomaterials such as gelatin methacryloyl (GelMA) serve as effective scaffolds supporting the in vitro expansion and in vivo transplantation of corneal endothelial cells and thus facilitate the reconstruction of the endothelial monolayer [29].

## 3 Key Hydrogel Properties for Corneal Applications

### 3.1 Biocompatibility

In the corneal applications of hydrogels, biocompatibility is a critical prerequisite of successful clinical translation. The essential requirements of biocompatibility include low cytotoxicity, low immunogenicity, and the ability to support cell growth and tissue integration. Hydrogels composed of natural polysaccharides and proteins, such as chitosan, collagen, and gelatin, demonstrate negligible cytotoxicity while effectively promoting the proliferation and migration of corneal epithelial cells [10, 30, 31]. Traditionally, corneal perforations are treated using cyanoacrylate glue as a temporary sealant. However, the hydrolysis of cyanoacrylate glue releases potentially toxic compounds (such as formaldehyde and alkyl cyanoacrylates) capable of inducing corneal scarring and neovascularization, which often necessitates corneal transplantation [32]. McTiernan et al. developed an acellular liquid hydrogel—LiQD Cornea—that undergoes spontaneous gelation at body temperature and exhibits low cytotoxicity and immunogenicity, thus serving as a potential alternative to conventional corneal transplantation [32]. Given that pure‐polysaccharide hydrogels have limited biomedical applications because of their inadequate mechanical properties, various synthetic crosslinking agents have been explored to address this limitation. However, residual toxicity, harmful degradation byproducts, and the use of toxic organic solvents during processing hinder the realization of nontoxic biomaterials. The use of hazardous crosslinkers in hydrogel fabrication causes serious environmental, impurity, and toxicity concerns. Therefore, selecting cost‐effective, environmentally friendly, nontoxic, and biocompatible crosslinking agents is critical for hydrogel manufacturing [33]. Owing to their excellent cell‐supporting properties, hydrogels not only maintain the normal physiological structure of the cornea but also promote the adhesion, proliferation, and functional expression of corneal epithelial and stromal cells. To enhance the biocompatibility of hydrogels, priority should be given to developing low‐immunogenicity materials, avoiding toxic crosslinking agents, and adopting dynamic crosslinking designs. Two additional essential strategies involve optimizing the mechanical properties to match those of different corneal layers and improving material permeability to ensure normal oxygen and nutrient exchange in the cornea.

### 3.2 High Transparency and Optical Performance

The high transparency and excellent optical properties of hydrogels make them perfect biomaterials for corneal repair, artificial corneal substitutes, and contact lens applications. Through optimization, hydrogels can achieve a visible light transmittance of >94%, which matches the optical properties of the natural cornea [34]. The refractive index of hydrogels must be very close to that of the natural cornea (∼1.376) or tunable within this range [35]. The cornea precisely regulates its hydration state to maintain transparency, a phenomenon fundamentally explained by Maurice's lattice theory, which posits that the uniform diameter and regular spacing of collagen fibrils enable the destructive interference of scattered light [36]. Modern hydrogel design aims to replicate this structural regularity or achieve amorphous homogeneity to minimize light scattering, with unique orderly structures and cellular mechanisms serving as the foundation for preserving optical clarity and visual function [37]. Damage to the corneal endothelial function can lead to edema and vision impairment [18].

### 3.3 Sufficient Mechanical Strength

The corneal surface is subjected to eyelid friction and intraocular pressure. Hence, the corresponding hydrogels must possess excellent mechanical strength, including resistance to tensile, compressive, and tear forces. To achieve scarless regeneration, the mechanical properties of hydrogels designed for corneal defect repair should match those of the natural corneal tissue. Robust nanoengineered hydrogels with strengths ranging from the kPa to MPa level can be obtained by integrating carboxylated single‐walled carbon nanotubes into chitosan and collagen [38]. The mechanical strength of hydrogels can be modulated by changing the crosslinking strategy, for example, shifting from a single crosslinking type to a dual‐ or triple‐network structure can optimize the balance between rigidity and flexibility [39]. Additionally, crosslinking by multiple ions (e.g., Fe^3+^ and Ca^2+^) can notably enhance hydrogel strength. Metal‐ion crosslinking can increase the compressive strength of hydrogels to 5.56 MPa, a value far surpassing that of hydrogels produced through traditional nonionic crosslinking [40].

### 3.4 Strong Adhesion in Hydrated Environments

The corneal surface is covered by a tear film, and hydrogels must achieve stable adhesion in this hydrated environment to ensure good integration and prevent displacement or detachment. Leveraging the principle of electrostatic attraction, positively charged hydrogels can bind to the negatively charged mucin layer on the corneal surface. Cationic peptide–based hydrogels achieve enhanced retention and adhesion on the hydrated corneal surface through ionic interactions with mucins, demonstrating adhesive performances superior to those of anionic peptide–based hydrogels in moist environments [41]. Photocrosslinking can improve the adhesive strength of hydrogels on the corneal surface. A hydrogel based on GelMA and oxidized dextran was reported to experience rapid crosslinking via photoinitiation on the hydrated corneal surface, achieving high adhesive strength and resistance to enzymatic degradation. Specifically, this hydrogel achieved stable sutureless adhesion for 56 days in a rabbit corneal transplantation model [12]. Hydrogels with dual covalent crosslinking (achieved by Schiff base reactions combined with photopolymerization) can form robust adhesive interfaces in physiological wet environments. This structural design addresses the insufficient long‐term wet adhesion stability of traditional adhesives, thereby satisfying the requirements for extended‐duration corneal repair in transplantation applications [42]. To address the challenge of bonding rigid hydrogels at wet interfaces, Koo et al. have developed a split‐brushing adhesion method that achieves a high adhesion energy, demonstrating a 67.5‐fold enhancement compared with conventional topological adhesion techniques. This approach is particularly suitable for integrating rigid hydrogel components for corneal repair [43]. Inspired by mussel underwater adhesion, Wang et al. synthesized a photocurable hydrogel by combining dopamine‐modified methacrylated hyaluronic acid (HA) with polyether F127 diacrylate. This material enables the sutureless sealing of full‐thickness corneal wounds through the covalent crosslinking of its catechol groups with proteins in the hydrated corneal tissue [44]. By optimizing chemical bonding, designing microstructures, and employing biomimetic strategies, one can obtain hydrogels that effectively meet adhesion requirements in the moist corneal environment while fulfilling cornea‐specific criteria such as transparency and biocompatibility [45].

### 3.5 Low Swelling Ratio and Morphological Stability

Hydrogels intended for corneal applications must maintain a stable volume in aqueous environments. Excessive swelling may induce edema, compromised wound sealing, irregular corneal curvature, and even damage to the corneal tissue [46]. Hydrogels with negligible swelling behavior effectively promote cell integration and wound repair [12, 47]. Therefore, swelling control is a critical consideration in the design of corneal hydrogels to ensure their reliability and therapeutic efficacy.

Swelling can be effectively suppressed by increasing the crosslinking density of the polymer network. By modulating the carboxymethyl chitosan to sodium alginate mass ratio, Lv et al. adjusted the swelling ratio of the hydrogel in the wide range of 1–450 [48]. Swelling in composite hydrogels can be further reduced by reinforcing the crosslinked network [49]. In conventionally designed hydrogels, the cleavage of polymer networks may cause considerable morphological changes, namely degradation‐induced swelling. By modulating the polymer–solvent interaction, hydrogels can achieve zero expansion under physiological conditions while maintaining their original morphology [50].

### 3.6 Rapid In Situ Gelation

In situ‐formed hydrogels are injectable or spreadable materials that undergo rapid gelation upon application to the corneal surface or defects, which affords a stable 3D network structure. Besides corneal repair, these hydrogels can serve as drug carriers to overcome the low bioavailability and corneal barrier limitation of traditional eye drops, increasing the drug retention time and reducing the side effects associated with frequent administration [51]. Thermosensitive or photocrosslinkable hydrogels can rapidly gel in situ under stimulation by body temperature or light exposure and can be thoroughly mixed with drugs in their low‐temperature liquid state, applied to the cornea, undergo in situ gelation, and slowly release the drug [52, 53]. The advantages of this approach include minimal invasiveness, biocompatibility, and multifunctionality, providing an integrated strategy for corneal repair from structural replacement to functional reconstruction.

### 3.7 Biodegradability and Safety

Hydrogels designed for corneal applications must exhibit controlled biodegradability. The degradation mechanism of hydrogels should align with the corneal healing cycle to prevent long‐term residue persistence and subsequent complications [54]. The degradation rate can be modulated by polymer and crosslinking strategy selection. Natural polymers generally demonstrate superior biodegradability and lower toxicity than synthetic ones. In particular, starch‐based hydrogels have been extensively studied owing to their inherent biodegradability and nontoxic nature [55]. Hydrogels for corneal tissue engineering should possess sustained‐release degradation profiles and enhanced mechanical properties to support the repair of stromal defects and ensure that hydrogel degradation does not interfere with the critical phases of tissue regeneration [56]. In summary, the biodegradability of hydrogels in corneal applications must be precisely controlled and the degradation byproducts should be nontoxic to ensure adequate mechanical support and avoid foreign body reactions.

### 3.8 Sterilization Strategies for Clinical Translation

Sterilization is a critical prerequisite for the clinical translation of corneal hydrogels. Inadequate sterilization can lead to postoperative infections, while inappropriate methods may compromise material structure, biocompatibility, and functionality. Owing to their proteinaceous or polysaccharide nature, natural hydrogels are particularly sensitive to conventional sterilization techniques, with heat and radiation‐based methods being potentially destructive [57]. Although synthetic hydrogels often exhibit higher tolerance, they may still suffer from chain scission or reduced mechanical stability under harsh conditions [58].

Traditional steam sterilization induces the irreversible denaturation of proteins and hydrolysis of polysaccharides, thereby destroying the triple‐helical structure and mechanical integrity of hydrogels [57]. UV sterilization is a low‐cost nontoxic surface sterilization method suitable for hydrogel contact lenses or surface implants. UV radiation effectively inactivates microorganisms but has a limited penetration depth and is therefore unsuitable for sterilizing thick hydrogel scaffolds [58]. Ethylene oxide sterilization is suitable for natural hydrogels sensitive to heat and humidity. However, residual ethylene oxide and related byproducts (such as ethylene chlorohydrin) must be thoroughly removed via aeration to ensure biocompatibility [58]. Gamma irradiation and electron beam sterilization are widely applied to synthetic hydrogels because of their high penetration ability and broad antimicrobial spectrum but may induce hydrogel oxidation and crosslink degradation. Hence, the parameters of these treatments must be optimized to prevent material damage [59]. Filtration sterilization is applicable to injectable hydrogel precursors with a particle size below 0.22 µm. This method avoids chemical or radiation‐induced damage but is limited to liquid formulations and cannot be applied to preformed hydrogel scaffolds. For preformed scaffolds, supercritical carbon dioxide (sc‐CO_2_) sterilization offers a promising nonthermal alternative. sc‐CO_2_ exhibits high penetrability and enables effective microbial inactivation while preserving the bioactive moieties and ultrastructure of sensitive scaffolds such as decellularized ECM (dECM) and collagen [60].

Future material design must factor in sterilization compatibility early in the development phase to bridge the gap between benchtop research and clinical applications.

## 4 Classification and Characteristics of Hydrogels

### 4.1 Natural Hydrogels

Natural hydrogels are based on proteins, polysaccharides, and dECM and are widely used in corneal repair, regeneration, and drug delivery owing to their excellent biocompatibility, light transmittance, and biodegradability. As their compositions are similar to that of the corneal ECM, these hydrogels support the adhesion, proliferation, and migration of corneal epithelial cells and stromal keratocytes [13, 47]. In addition, natural hydrogels can mimic the function of the corneal EBM by delivering growth factors or anti‐inflammatory agents to inhibit fibrosis and reduce scar formation [23]. Some natural hydrogels demonstrate a visible light transmittance of over 94% while maintaining long‐term transparency, meeting the stringent optical requirements of corneal tissue [34]. However, natural hydrogels often exhibit poor mechanical properties because of their low crosslinking density, which limits their ability to withstand prolonged mechanical stress [61]. To address this drawback, reinforcement strategies such as compositing with synthetic materials or dual‐crosslinking have been explored [56]. The bioactive components of natural hydrogels may lose their functionality during high‐temperature or irradiation sterilization [61], while animal‐derived hydrogels can pose the risks of immunogenicity and batch‐to‐batch variability [62].

#### 4.1.1 Collagen Hydrogels

Aggregated collagen fibers form load‐bearing structures in connective tissues, including the corneal stromal lamellae [63]. Collagen accounts for 70% of the cornea's weight, with type I collagen (Col‐I) being most abundant, and is therefore widely used for constructing scaffolds that mimic the corneal composition [64]. In the cornea, collagen fibers (diameter: 25–35 nm) are densely packed and uniformly distributed across stromal layers to form orthogonal lamellae or sheets. Each lamella comprises ∼200 collagen fibrils aligned in parallel and perpendicular to the adjacent lamellae. The precise spatial arrangement of collagen fibrils underlies the mechanical strength and optical transparency of the cornea [65]. Ye et al. developed a collagen‐based hydrogel emergency contact lens loaded with acidic fibroblast growth factor for treating corneal injuries [66]. This lens combines macromolecular collagen with polymeric materials, preserving the bioactivity and structural stability of the constituents while enabling the delivery of various unstable proteins, and exhibits anti‐inflammatory and antifibrotic properties, demonstrating efficacy in treating corneal alkali burns. Wu et al. developed a glycerol‐induced collagen‐based hydrogel that forms a lamellar structure at the macroscopic level, with the optical properties depending on the fibril diameter and spacing. This approach yielded an artificial corneal substitute with optimal optical clarity, mechanical strength, high permeability, manufacturability, ease of preservation, and in vitro biocompatibility, thus representing a green, simple, and effective strategy for the development of biomimetic artificial corneas [67].

Owing to their high homology with native corneal stromal components, collagen hydrogels are suitable for corneal stromal regeneration and epithelial layer reconstruction and can serve as the core layers of composite artificial corneal scaffolds [66]. These hydrogels exhibit exceptional biocompatibility and tissue integration capabilities, particularly in the repair of mild‐to‐moderate corneal stromal defects [65, 67]. Key limitations include insufficient mechanical strength in the natural state, immunogenicity risks associated with xenogeneic collagen sources, and difficulty in securing batch stability during large‐scale fabrication [44, 67, 68]. Recent advances, such as composite modification with silk fibroin or chitosan and UV crosslinking technology, have markedly enhanced the mechanical properties and degradation controllability of collagen hydrogels, expanding their potential applications in full‐thickness corneal defect repair [69].

#### 4.1.2 Gelatin Hydrogels

Gelatin is produced by the partial hydrolysis of collagen and, depending on the hydrolysis process, is classified as Type A (acid‐treated) or Type B (alkali‐treated), exhibiting enhanced water solubility posthydrolysis. The structure of gelatin can be simplified as (Gly–X–Y)_n_, where Gly represents glycine, and X and Y are typically proline or hydroxyproline [70].

Gelatin hydrogels exhibit transparency and low swelling rates comparable with those of the natural cornea and are therefore suitable for corneal defect repair while ensuring postoperative visual clarity [10]. However, traditional gelatin hydrogels exhibit limited resistance to swelling and degradation. The hydrogen bond–dominated interchain interactions may lead to uncontrolled mechanical properties, compromising stability in the dynamic corneal environment [71]. Adjustments in crosslinking density or the incorporation of functional materials (e.g., chondroitin sulfate aldehyde and silicate nanoparticles) are necessary to mimic the mechanical properties of native corneal tissue and thus address the above problems [72, 73]. In some studies, the degradation rate of gelatin hydrogels did not align with the temporal progression of corneal repair. Excessively rapid degradation can cause premature scaffold failure, whereas slow degradation may impede cell migration and tissue remodeling [74, 75]. Strategies such as the incorporation of enzyme‐responsive moieties or dynamic crosslinking designs have been explored to ensure controlled degradation [76, 77].

Although gelatin itself exhibits excellent biocompatibility, residual photoinitiators or crosslinking agents from the photocuring process may trigger inflammatory responses and thus adversely affect corneal transparency [12]. Additionally, highly crosslinked gelatin hydrogels may inhibit the migration of corneal epithelial cells, leading to delayed wound healing [78].

Gelatin hydrogels are widely applied in temporary corneal epithelial defect repair, drug delivery systems, and 3D bioprinting bioinks, particularly in superficial corneal injury treatment and postoperative adjuvant repair, owing to their favorable optical transparency, tunable degradability, and cost‐effectiveness [44, 79, 80]. Key limitations include high swelling ratios, poor mechanical stability in the native form, and rapid degradation that fails to meet the requirements for long‐term tissue support [13, 81]. Methacryloyl modification and dual crosslinking strategies (e.g., ionic crosslinking combined with photocrosslinking) allow one to effectively regulate hydrogel mechanical strength and degradation rate while retaining excellent biocompatibility, thereby enhancing competitiveness in the field of corneal stromal regeneration scaffolds [82, 83, 84].

#### 4.1.3 Fibrin Hydrogels

Fibrin—a provisional matrix naturally formed during wound healing—is highly compatible with the ECM and supports cell migration, adhesion, proliferation, and differentiation, thus being an excellent biomaterial with favorable biocompatibility and having widespread applications as a wound sealant and raw material for natural hydrogels [85]. Fibrin hydrogels are primarily formed through an enzymatic reaction between fibrinogen and thrombin. Thrombin cleaves fibrinogen to release fibrin monomers, which subsequently assemble into a 3D fibrous network structure [86]. In addition to featuring outstanding biocompatibility and superior cell adhesion properties, fibrin hydrogels possess a uniform highly porous structure. This architecture increases the surface area, facilitates cell–cell interactions, promotes angiogenesis, and enhances the absorption of nutrients and elimination of metabolic waste [87]. Fibrin hydrogels also demonstrate a high water absorption capacity and favorable swelling rates, enabling the rapid absorption of wound exudates while maintaining a moist wound environment to accelerate healing and protect the wound site [88]. Furthermore, fibrin exhibits anti‐inflammatory and antioxidant properties, creating a microenvironment conducive to tissue regeneration and wound repair.

However, natural fibrin gels have a low mechanical strength, which limits their ability to repair high‐load‐bearing tissues. Reinforcement strategies such as compositing with rigid materials or constructing double‐network structures have been explored [89]. Additionally, the traditional thrombin‐driven gelation process lacks precise control over fibrin network formation, which may cause batch‐to‐batch variability [85]. The large‐scale production of fibrin hydrogels is also hindered by high costs and difficulties in sterilization [90].

As derivatives of the natural coagulation matrix, fibrin hydrogels possess excellent biocompatibility and cell adhesion capability and are therefore ideal for temporary sealing, hemostasis, and the early inflammatory regulation of superficial corneal trauma [12, 85, 91]. These hydrogels provide unique advantages in the emergency treatment of corneal perforations and adjuvant therapy for postoperative wound healing. Key limitations include extremely low mechanical strength, rapid degradation that prevents long‐term tissue support, high large‐scale production costs, and sterilization difficulties [23, 89, 92].

#### 4.1.4 Silk Fibroin Hydrogels

Silk fibroin is a protein primarily comprising light and heavy chains in a 1:1 ratio linked via disulfide bonds and exhibits unique amphiphilic characteristics due to the coexistence of hydrophobic (ordered and highly conserved) and hydrophilic (less ordered and relatively complex) structural blocks. This structure endows silk fibroin with excellent elasticity and toughness, enabling exceptional performance in diverse applications [93].

Silk fibroin hydrogels are highly transparent and therefore readily meet the optical requirements of the cornea. Ghosh et al. developed a silk fibroin hydrogel by introducing a photoinitiation system to form dityrosine crosslinks, simultaneously achieving outstanding transparency and soft elastic properties and thereby fulfilling the demands of corneal tissue engineering [94]. The incorporation of methacrylated silk fibroin into decellularized corneal stroma can further enhance the mechanical properties of composite hydrogels. However, highly concentrated silk fibroin solutions tend to form β‐sheet crystals, which may compromise transparency or cytocompatibility and necessitate the suppression of excessive crystallization through preoxidation or nanofibrillation [95, 96]. The poor mechanical performance of pure‐silk‐fibroin hydrogels can be remediated by adding a suitable amount of other polymers (e.g., polyacrylamide and chitosan) or incorporating nanofiber reinforcements to match the elastic modulus and tensile strength of the corneal stroma [97, 98]. As silk fibroin inherently lacks cell‐adhesive motifs, it also requires other components (e.g., collagen and gelatin) or chemical modifications (e.g., methacrylation) to improve cell adhesion and bioactivity [99, 100]. Although silk fibroin generally demonstrates low immunogenicity, residual fragments after long‐term implantation may induce chronic inflammation, which highlights the need for further in vivo safety validation [101, 102].

The high transparency, excellent mechanical properties, and biocompatibility of silk fibroin hydrogels make them promising materials for artificial corneal substitutes, corneal stromal regeneration scaffolds, and smart wearable devices [25, 103]. These hydrogels perform exceptionally well in full‐thickness corneal defect repair and long‐term implantable corneal devices. Key limitations include reduced transparency due to excessive β‐sheet crystallization in high‐concentration solutions, lack of inherent cell‐adhesive motifs, and potential chronic inflammatory responses induced by residual fragments after long‐term implantation [104, 105]. Modifications such as methacryloylation, composite blending with HA, or the incorporation of antimicrobial peptides can simultaneously enhance cell compatibility, antibacterial performance, and structural stability, facilitating clinical translation [94, 95].

#### 4.1.5 HA Hydrogels

As a linear polysaccharide, HA is composed of repeating disaccharide units of d‐glucuronic acid and *N*‐acetylglucosamine linked by β‐1,3‐glycosidic bonds; these units form long chains via β‐1,4‐glycosidic bonds. Given that HA is a key component of the ECM [106] and ubiquitously present in the cornea [107], HA hydrogels can closely mimic the biochemical properties of the ECM [108]. Owing to its high hydrophilicity and extended chain length, HA exhibits remarkable water‐binding capacity. HA hydrogels achieve visible‐light transmittances (>94%) close to that of the natural cornea (∼97%) [34]. The similarity between the refractive index of these hydrogels (1.336–1.347) and that of the corneal tissue (1.376) minimizes visual disturbances caused by light scattering, which is critical for vision restoration [10, 34]. HA hydrogels possess strong formability and can rapidly establish a stable 3D network structure within minutes through photocrosslinking, enzymatic crosslinking, or ionic crosslinking [53, 109]. Shen et al. developed a composite hydrogel composed of methacrylated HA and decellularized corneal stroma, revealing its ability to directly fill corneal defects of varying shapes without requiring sutures while maintaining long‐term stable adhesion [56]. HA hydrogels facilitate corneal epithelial cell migration and stratification by mimicking the EBM structure. Kang et al. developed a bioorthogonally crosslinked HA hydrogel incorporating growth factor–tethered photocleavable bonds to accelerate corneal regeneration, establishing a positive correlation between the epithelial regeneration rate and the frequency of UV irradiation [110]. The viscoelasticity and bioadhesive properties of HA make it an ideal drug delivery vehicle. Yao et al. engineered a nerve growth factor–loaded HA hydrogel to obtain a sustained‐release drug delivery system on the corneal surface, extending the drug retention time more than fourfold [111]. In physiological environments, unmodified HA hydrogels degrade within only two weeks. The addition of polyethylene glycol (PEG) can prolong the degradation period to several weeks but may compromise cellular biocompatibility [109]. Additionally, certain bioorthogonal or photocrosslinking strategies for HA hydrogels rely on specific conditions (e.g., UV light and catalysts), potentially increasing surgical complexity. For instance, photocurable hydrogels require the precise control of irradiation duration and intensity to minimize phototoxicity to corneal cells [110].

HA hydrogels closely mimic the biochemical properties of the corneal ECM and exhibit excellent optical transparency and biocompatibility, thus finding numerous applications in corneal epithelial repair, sustained drug delivery systems, and sutureless corneal defect repair and exhibiting remarkable efficacy in the treatment of chemical burns and dry eye–related corneal injuries [47, 112, 113]. Critical limitations include the rapid degradation of unmodified HA, insufficient mechanical strength, and potential cytotoxicity associated with certain crosslinking strategies [47, 53, 114]. Composite modification with decellularized corneal matrix or the adoption of bioorthogonal crosslinking technology enables the precise matching of the degradation rate with the corneal healing cycle while improving mechanical stability [106, 115].

#### 4.1.6 Chitosan Hydrogels

Chitosan is a copolymer composed of *N*‐acetyl‐d‐glucosamine and d‐glucosamine units [116] and obtained through the deacetylation of chitin. Chitin is commercially derived from crustacean waste generated during seafood processing [117]. Chitosan hydrogels exhibit high transparency (transmittance > 85%) and optical properties compatible with those of the cornea. Notably, chitosan has antimicrobial properties. Yang et al. developed an electrochemically crosslinked chitosan hydrogel contact lens with excellent optical performance, mechanical properties, biocompatibility, and an antiadhesive efficacy against *Staphylococcus aureus* (*S. aureus*) comparable with that of commercial contact lenses [118]. Jiao et al. constructed an antibacterial and antioxidant contact lens using a hydrogel composed of quaternized chitosan and tannic acid. Quantitative and qualitative antibacterial assays against *S. aureus* and *Escherichia coli* (*E. coli*) demonstrated the superior bactericidal efficacy of this hydrogel, particularly against *E. coli* (nearly 100%) [119]. Chitosan hydrogels have also shown efficacy in the treatment of infectious corneal diseases. However, the inherent antimicrobial activity of pure chitosan hydrogels may be insufficient to suppress the proliferation of multidrug‐resistant strains, and supplementary antibacterial components may therefore be required [119, 120]. Huang et al. developed a hydrogel that comprised quaternized chitosan, silver nanoparticles, and graphene oxide and enabled the sustained release of antifungal drugs. This system enhanced local drug bioavailability while minimizing the side effects associated with conventional treatments such as eye drops or intravitreal injections [121].

Although chitosan is an ideal material for wound dressings, pure‐chitosan dressings exhibit strong adhesion to the corneal surface and may therefore damage the newly regenerated epithelial cells upon removal. Formulating chitosan into hydrogels enables on‐demand dissolution and reduces the risk of scar formation [122]. Currently, 3D printing technology for chitosan hydrogels remains underdeveloped, and the precise replication of the intricate microstructure of biomimetic corneas (e.g., lamellar collagen alignment) is challenging. Existing printing methods also face limitations in resolution and cell‐loading capacity [123, 124].

The inherent antimicrobial activity, favorable tissue adhesion, and biocompatibility of chitosan hydrogels make them suitable for the treatment of infectious keratitis, corneal wound dressings, and drug carriers [118, 125, 126, 127]. These hydrogels demonstrate advantages in the repair of corneal ulcers caused by bacterial infections and superficial defects [119]. Major limitations include poor solubility under physiological pH conditions, insufficient mechanical strength, and difficulty in batch standardization during large‐scale production [128, 129]. Quaternization modification and composite blending with silver nanoparticles or graphene oxide can notably enhance the antimicrobial activity, water solubility, and mechanical properties of chitosan hydrogels, expanding their applications in the treatment of complex corneal injuries [119, 121].

#### 4.1.7 Alginate Hydrogels

Alginate is an anionic polysaccharide extracted from brown algae and certain bacteria, finding numerous applications owing to its unique gelation properties, biocompatibility, and tunable physicochemical characteristics [130]. This polysaccharide is a linear copolymer composed of β‐d‐mannuronic acid (M) and α‐l‐guluronic acid (G) residues linked by 1,4‐glycosidic bonds. The physicochemical properties of the corresponding hydrogels, such as gelation capability, mechanical strength, and biodegradability, are critically governed by the M/G ratio and the sequence of the M and G units. The M/G ratio strongly depends on the biological source of alginate [130, 131, 132].

Alginate hydrogels exhibit excellent cell affinity, and their 3D porous structure provides a biomimetic microenvironment for cells. Zhang et al. developed a dual‐network hydrogel composed of alginate and nanoscale spherical dendrimers. This hydrogel featured a moderate pore size and swelling properties, demonstrating outstanding biocompatibility, tunable mechanical performance, and adhesive characteristics, and thus offering a novel approach to sutureless corneal transplantation [133]. Alginate can also enhance the ocular bioavailability of nanomedicines. Ger et al. developed an alginate‐coated nanoceria eye drop formulation in which an alginate coating improved the adhesion of metallic nanocarriers, prolonged their retention time in the anterior chamber, and increased their surface stiffness, thereby promoting their cellular uptake and enhancing their bioactivity. In an animal model of corneal abrasion, this formulation markedly reduced the area of corneal epithelial injury [134]. Porosity is another critical factor that influences drug release from alginate hydrogels. Siboro et al. fabricated porous and nonporous alginate hydrogels and demonstrated that porosity accelerated drug release, whereas the nonporous hydrogels released the drug very slowly. Specifically, porous alginate hydrogels sustainably released doxorubicin for up to 35 days, offering a novel strategy for ocular surface drug delivery [135]. Although porous structures can prolong the duration of drug release, very high porosity compromises mechanical strength, whereas drug release from nonporous structures is very slow [136]. Hence, a trade‐off between porosity and mechanical properties is present.

Alginate hydrogels are biocompatible, injectable, and ion‐crosslinkable, thus being suitable for corneal defect filling, cell transport, and temporary wound sealing [134, 137, 138, 139]. These hydrogels can be conveniently applied in the repair of irregular corneal defects and minimally invasive surgeries [140]. Key limitations include moderate mechanical strength, uncontrollable degradation rates in mammals, and insufficient cell adhesion sites [141]. The construction of double‐network structures or composite modification with cell‐adhesive peptides can enhance the mechanical properties and cell compatibility of alginate hydrogels while enabling the precise regulation of degradation rates [136, 142].

#### 4.1.8 Cellulose Hydrogels

Cellulose, the most abundant natural polymer, is composed of linear chains of glucose residues linked by β‐1,4‐glycosidic bonds, the corresponding primary sources being woody plants, herbs, flax, cotton, and bacteria [62, 143]. Cellulose and its derivatives (e.g., nanocellulose) exhibit inherent biocompatibility, facilitating the adhesion and proliferation of corneal epithelial cells. Luo et al. developed a cellulose hydrogel by integrating cellulose nanocrystals with poly(2‐hydroxyethyl methacrylate). This hydrogel mimicked the lamellar helical structure of the native cornea and optimized the cellular microenvironment to support corneal tissue regeneration, thereby offering a viable strategy for rapid corneal repair [144]. The optical transparency of cellulose hydrogels is a critical advantage in corneal applications. Hu et al. fabricated a cellulose hydrogel using an aqueous NaCl regeneration method and achieved a transparency (>94%) close to that of the natural cornea [145]. The mechanical properties of cellulose hydrogels can be precisely modulated by chemical crosslinking or physical modification. Li et al. engineered a hybrid cellulose/polyacrylamide hydrogel via the efficient multiscale dissolution of cellulose and in situ polymerization of polyacrylamide. This hydrogel demonstrated excellent tensile strength, compressive resistance, conductivity, and ultralong cycling stability, thus being suitable for human–machine interaction applications and inspiring novel designs for corneal sensors [146]. As a renewable natural polymer, cellulose has low toxicity and is environmentally friendly. Yin et al. developed a multifunctional conductive hydrogel using sodium carboxymethyl cellulose and an MXene (MXenes are a class of two‐dimensional transition metal carbides, nitrides, and carbonitrides). This hydrogel combined superior mechanical properties with electrical conductivity and biosafety and was therefore deemed suitable for use in flexible electronics and long‐term implants [147]. However, the performance of cellulose hydrogels (e.g., porosity and swelling ratio) strongly depends on raw material purity, crosslinking methods, and processing conditions, which may lead to batch‐to‐batch variability and pose challenges for large‐scale production [148].

As renewable natural polymers, cellulose hydrogels exhibit high transparency, excellent mechanical strength, and biocompatibility, thus being suitable for use in corneal repair scaffolds, smart sensors, and wearable devices [149, 150] and holding promise for corneal injury repair and ocular physiological signal monitoring [144, 147]. Major limitations include complex fabrication processes, swelling behavior that compromises structural stability, and performance fluctuations caused by the aggregation of nanocellulose [151, 152, 153].

### 4.2 dECM Hydrogels

dECM hydrogels are derived from natural ECM components sourced from porcine, human, or other mammalian corneal tissues. Following decellularization to remove immunogenic components, these hydrogels retain essential ECM constituents such as collagen, glycosaminoglycans, and glycoproteins [154, 155]. Owing to their ability to replicate the biochemical and structural properties of the native corneal ECM, dECM hydrogels hold promise for corneal repair and regeneration, facilitating corneal cell migration, proliferation, and tissue remodeling while demonstrating excellent biocompatibility and tissue integration. Crosslinking strategies enable the precise tailoring of hydrogel mechanical strength, degradation rate, and transparency to meet the optical clarity and biomechanical stability requirements of the cornea. Yazdanpanah et al. developed a photocrosslinkable porcine corneal dECM hydrogel with controlled swelling behavior. This hydrogel maintained high transparency while exhibiting mechanical properties comparable with those of the native corneal tissue and could be crosslinked in situ under visible light, which resulted in notable biomechanical strength, stability, and adhesive property enhancement [156]. Additionally, dECM hydrogels can be directly applied to irregular corneal defects via injection or filling, adapting to diverse lesion morphologies and promoting tissue regeneration without the need for sutures and thereby minimizing surgical trauma [56]. These hydrogels support the maintenance of corneal cell–specific phenotypes; replicate the ultrastructure, protein composition, and optical properties of the human cornea; and facilitate corneal stromal reconstruction after injury by sustaining cell viability, proliferation, and keratin expression [83]. However, the inherent mechanical weakness of pure dECM hydrogels limits their applicability as bioinks for 3D bioprinting [157] and necessitates the further optimization of crosslinking approaches. Moreover, xenogeneically derived dECM materials may pose immunological risks, requiring a comprehensive preclinical evaluation of safety and efficacy as well as standardized manufacturing protocols [158].

dECM hydrogels can precisely mimic the biochemical composition and structure of the native corneal ECM and are therefore ideal for corneal regeneration and repair [83]. These hydrogels are particularly suitable for full‐thickness corneal defect repair and the fabrication of artificial corneal scaffolds and 3D bioprinting bioinks, offering considerable advantages in clinical translation [56, 156]. Key limitations include insufficient mechanical strength, immunological risks associated with xenogeneic sources, and batch‐to‐batch variations caused by complex decellularization processes [157, 158, 159].

### 4.3 Synthetic Polymer Hydrogels

Since the seminal work of Wichterle and Lim (1960), who introduced crosslinked poly(2‐hydroxyethyl methacrylate) (PHEMA) as the first synthetic hydrogel for contact lenses, synthetic polymers have revolutionized ocular biomaterials [160]. Unlike natural polymers, synthetic hydrogels offer exceptional batch‐to‐batch consistency and widely tunable mechanical properties.

#### 4.3.1 PEG Hydrogels

PEG, a polyether composed of repeating ethylene oxide units, is characterized by high hydrophilicity, biocompatibility, and chemical inertness. With a broad molecular weight range of 300–10 000 000 g mol^−1^, PEG displays properties suitable for various applications [161]. In particular, the mechanical properties of PEG hydrogels can be precisely modulated to meet the requirements of corneal tissue engineering by adjusting the crosslinker ratio, polymer composition, and photopolymerization conditions. By modifying the structure and chemical composition of crosslinkers, one can tailor the degradation rate and elastic modulus of these hydrogels to match the biomechanical properties of the corneal stroma [162, 163]. However, the swelling behavior of these hydrogels may compromise their stability, necessitating the optimization of crosslinker density or the introduction of a double‐network structure to counteract swelling [162]. PEG hydrogels also exhibit excellent cytocompatibility, supporting the survival and proliferation of corneal epithelial and stromal cells [164]. However, pure‐PEG hydrogels lack bioactive signals inherent to the native ECM and therefore require functionalization with additional bioactive molecules to promote cell adhesion and proliferation [165]. PEG hydrogels can also serve as drug delivery vehicles for the locally sustained release of anti‐inflammatory or antiangiogenic agents. Xu et al. developed a PEG hydrogel loaded with antivascular endothelial growth factor to notably inhibit corneal neovascularization and achieve sustained drug release for up to 28 days [166]. Although PEG hydrogels possess low immunogenicity, the potential formation of anti‐PEG antibodies may affect their long‐term applicability. Rare cases of PEG hypersensitivity have also been reported. To mitigate immunogenicity and enhance functionality, PEG can be combined with other biomaterials such as silk fibroin or chitosan to form composite hydrogels [167].

PEG hydrogels exhibit excellent optical transparency, biocompatibility, and structural tunability, thus being suitable for drug delivery systems, corneal tissue engineering scaffolds, and smart responsive implants [168, 169, 170], and are widely used in antiangiogenic therapy and precise drug delivery [166, 171]. Key limitations include the lack of inherent cell‐adhesive motifs, potential anti‐PEG antibody responses after long‐term implantation, and the insufficient mechanical strength of pure‐PEG hydrogels [167, 172, 173].

#### 4.3.2 Polyvinyl Alcohol (PVA) Hydrogels

PVA is a water‐soluble polymer that is synthesized through the alcoholysis or hydrolysis of polyvinyl acetate and is rich in hydroxyl groups. The regular linear structure of PVA facilitates the formation of an ordered array via intramolecular hydrogen bonds, endowing the polymer with excellent hydrophilicity, film‐forming capability, adhesion, and biocompatibility [174, 175]. The light transmittance of PVA hydrogels (>96%) is sufficiently close to that of the natural cornea. Liu et al. used a syneresis‐salting‐out synergistic crystallization strategy to develop a strong and lubricious PVA hydrogel with a visible‐light transparency of 98%. Contact lenses fabricated from this hydrogel satisfied optical imaging demands while offering favorable biocompatibility and sustained drug‐release functionality, thereby providing novel material design insights for ocular wearable devices [176]. PVA hydrogels also possess broadly tunable mechanical properties. Wu et al. reversibly and extensively modulated the mechanical properties of PVA hydrogels by adjusting the aggregation state of polymer chains via the Hofmeister effect. This approach enabled the simultaneous realization of high mechanical performance and wide dynamic adjustability in hydrogels, thus fulfilling the stress requirements of corneal applications [177]. Given that unmodified PVA hydrogels degrade rapidly and do not match the recovery cycle of corneal injuries, Wang et al. developed a novel double‐network hydrogel by combining fish‐derived collagen with PVA. The degradation rate of this hydrogel could be precisely controlled by adjusting the collagen‐to‐PVA ratio, and the hydrogel thus provided structural support for corneal regeneration after injury [178].

PVA hydrogels possess ultrahigh transparency, favorable mechanical strength, and biocompatibility, thus being preferred materials for artificial corneal substitutes, contact lenses, and corneal wound dressings [22, 176, 179, 180]. Major limitations include slow degradation or nondegradability, poor cell adhesion, and potential foreign body reactions after long‐term implantation [45, 181].

#### 4.3.3 Polyacrylamide Hydrogels

These hydrogels are formed by polymerizing acrylamide monomers into a network structure. Acrylamide forms polymer chains and provides fundamental mechanical strength and swelling capacity [182, 183]. The concentration of polyacrylamide during synthesis may influence its properties. Compared with the conventional 7.5% polyacrylamide concentration, a concentration of 18% can yield a stiffer polymer network with a higher mechanical strength [184]. However, pure‐polyacrylamide hydrogels may exhibit insufficient puncture resistance and strength, which necessitates the use of reinforcing materials such as TiO_2_ and carbon nanotubes [185]. Crosslinking density adjustment facilitates precise control over the mechanical properties of polyacrylamide hydrogels, allowing them to mimic the physiological stiffness of the corneal tissue. Norris et al. developed a photodegradable polyacrylamide hydrogel by copolymerizing acrylamide with a photocleavable *o*‐nitrobenzyl bisacrylate crosslinker, showing that this system enables the dynamic modulation of hydrogel stiffness via light intensity adjustment [186]. Light irradiation can also be used to control the degradation of polyacrylamide hydrogels. Li et al. introduced a crosslinked sodium polyacrylate hydrogel that rapidly degraded into a soluble form under UV light without requiring additional chemical reagents. The recycling efficiency of this method is nearly 200 times that of traditional deesterification‐based approaches, resulting in a notably reduced environmental impact [187]. Polyacrylamide hydrogels can serve as drug carriers, offering novel strategies for treating corneal infections and providing a platform for controlled drug release during corneal therapy [188, 189, 190]. However, severe infectious keratitis often requires multistage treatment, whereas existing polyacrylamide‐based hydrogels are mostly designed for single‐stage therapy. This discrepancy highlights the lack of comprehensive solutions in current approaches [13, 25].

Polyacrylamide hydrogels offer the advantages of tunable mechanical properties, high structural stability, and biocompatibility, thus being suitable for the fabrication of corneal sensors, drug carriers, and 3D printing scaffolds [53, 191, 192], as well as corneal physiological signal monitoring and the treatment of infectious keratitis [190, 193]. Key limitations include poor biodegradability, lack of cell‐adhesive motifs, and the potential cytotoxicity of high‐concentration materials [45, 194]. A comprehensive comparison of the properties, advantages, and limitations of these natural and synthetic hydrogels is presented in **Table** **1**.

**TABLE 1:** Comparative analysis of hydrogel materials for corneal applications: Properties, advantages, and limitations.

Material category	Material	Transparency (visible light)	Mechanical strength	Degradation	Advantages	Limitations	Refs.
Proteins	Collagen	High optical clarity (>90%) closely mimicking that of the native cornea	Native collagen hydrogels are weak (<10 kPa); crosslinking or compression can elevate stiffness to the MPa range (0.5–5 MPa) to match that of corneal stroma	Enzymatically degradable (collagenase); typically degrades over 1–3 months depending on crosslinking density	Can mimic the tissue structure and biological activity of the natural corneal stroma, supporting the regeneration of both corneal epithelium and stroma adjustable physicochemical and optical properties	Natural collagen hydrogels exhibit relatively weak mechanical properties, necessitating crosslinking or compounding with other materials to enhance their structural integrity collagen derived from different species may potentially induce immune responses remaining challenges in standardizing production processes, controlling costs, and achieving large‐scale fabrication	[44, 47, 66, 67, 79, 84, 195]
	Gelatin	High, >90% for GelMA	Highly tunable via crosslinking concentration adjustment; compressive modulus typically 10–300 kPa	Rapid degradation (weeks) under physiological conditions; modification (e.g., GelMA) is required to extend stability to 1–2 months for stromal support	Biocompatibility and cellular support optical transparency tunable mechanical properties	Insufficient adhesive strength uncontrollable degradation rate	[10, 12, 47, 196, 197, 198]
	Fibrin	Variable; often opaque or translucent; improves with thickness control	Low; elastic modulus is typically <10 kPa, inadequate for high‐load applications	Very rapid (days to <2 weeks) because of fibrinolysis; suitable for temporary sealants but not long‐term substitution	Facilitates wound healing biodegradability supports cell–matrix interactions	Insufficient mechanical strength difficulty in precisely regulating physicochemical properties unstable optical performance	[12, 56, 85, 86, 195, 199]
	Silk fibroin	High (>90%) when β‐sheet crystallization is controlled, matches native corneal refractive index (1.38)	Exceptional; tensile strength can reach 1–10 MPa	Slow and tunable; can persist for months to years, providing long‐term scaffolding	Biodegradability tunable mechanical properties transparency and structural designability promotes tissue regeneration and mimics the ECM	Pure silk fibroin exhibits insufficient mechanical strength may increase the risk of infection, requires the incorporation of antimicrobials or the optimization of the sterilization process slow gelation kinetics	[25, 94, 103, 200, 201, 202, 203, 204, 205]
Polysaccharides	HA	Superior (>95%); refractive index (1.33–1.35) is highly compatible with the ocular system	Native HA is a viscous fluid/weak gel; chemical modification increases storage modulus to 10–100 kPa	Fast (<2 weeks unmodified); tunable via crosslinking	Biocompatibility optical transparency matching that of corneal tissue tunable mechanical strength through chemical modification	HA hydrogels alone often exhibit insufficient mechanical strength and rapid degradation rates certain crosslinking agents or modifying components may induce localized inflammatory responses	[34, 47, 106, 110, 115, 195, 206, 207, 208, 209]
	Chitosan	Good (>85%–90%); highly dependent on deacetylation degree and pH	Tensile strength of 1–20 MPa for films or densely crosslinked hydrogels	Biodegradable by lysozyme; degradation rates vary from weeks to months	antimicrobial and anti‐inflammatory properties biocompatibility, optical transparency, and tissue adhesion	Limited solubility under physiological pH clinical translation complicated by difficulty in scaled‐up production, standardization, and quality control	[13, 118, 119, 121, 125, 129, 195, 210]
	Alginate	High (>90%); dependent on purity and M/G ratio	Moderate (kPa range); brittle at high concentrations, dual‐network strategies often required to improve toughness	Slow and uncontrollable in mammals because of the lack of alginase; relies on ion exchange dissolution	Hydrophilicity and cytocompatibility tunable mechanical and optical properties Injectable, facilitating clinical manipulation	Inadequate mechanical properties rapid degradation lack of cellular adhesion	[10, 56, 133, 206, 211, 212, 213, 214, 215, 216]
	Cellulose	High (>90% for nanocellulose)	High; tensile strength can exceed 10 MPa, closely matching or surpassing native corneal strength	Stable; nonbiodegradable in humans (requires modification)	Low toxicity and biodegradability supports corneal epithelial cell growth abundant and renewable sources	Complex fabrication process swelling/deswelling behavior may compromise stability susceptibility to colloidal aggregation	[47, 144, 145, 217, 218, 219, 220, 221, 222, 223, 224, 225]
Animal tissues	Decellularized corneal matrix	Can be restored to near‐native levels (>85%–90%) after gelation and remodeling	Lower than that of native cornea but improvable via photocrosslinking	Bioresorbable; participates in tissue remodeling	Bioactivity and biocompatibility structural biomimicry Injectability with in situ self‐repair capability	Possible batch‐to‐batch variation and pathogen risks insufficient mechanical properties costly decellularization processes limit widespread clinical adoption	[56, 79, 83, 156, 158, 226]
Synthetic polymers	PEG	Excellent (>95%); low light scattering	Highly tunable (kPa to MPa); dependent on molecular weight and crosslinker geometry	Nonbiodegradable backbone; degradation requires the incorporation of hydrolytically or enzymatically cleavable crosslinkers	Rapid gelation with operational convenience excellent optical properties tunable mechanical properties	Insufficient adhesion and long‐term stability limited bioactivity	[25, 32, 47, 83, 84, 166, 227]
	PVA	Superior (>98%); widely used in contact lenses	High (MPa range); capable of withstanding eyelid shear forces and intraocular pressure	Slow/nondegradable	Biocompatibility ability to mimic the mechanical properties of the cornea optical transparency	Inadequate cell adhesion limited bioactivity insufficient long‐term stability	[22, 25, 176, 228, 229, 230, 231]
	Polyacrylic acid	High (>90%); excellent optical transmission	High toughness and elasticity; can be engineered to resist puncture (MPa range)	Nonbiodegradable	Biocompatibility tunable mechanical properties	Nondegradability of certain hydrogels raises long‐term safety concerns Swelling behavior may lead to inadequate mechanical properties Challenges in scaling up production	[119, 144, 183, 185, 186, 232, 233, 234, 235]

## 5 Applications of Hydrogels in Corneal Scenarios

The functional design of hydrogels is evolving from single therapeutic purposes to synergistic multiscenario applications. For corneal injury repair, the porous structure of hydrogels mimics the natural ECM environment of the cornea, providing a temporary scaffold for epithelial cells to facilitate their migration and proliferation and thereby accelerating tissue regeneration [236, 237]. Although artificial corneas have not yet fully replaced traditional transplants, the excellent optical transparency, tunable mechanical properties, and biocompatibility of hydrogels make them promising corneal substitutes. The structural support offered by hydrogels can also guide collagen fiber alignment to replicate the natural corneal architecture, which is useful for research and translational applications [238, 239]. In corneal drug delivery, hydrogels enhance therapeutic efficacy by prolonging drug retention, improving drug penetration, and enabling stimuli‐responsive drug release. Their synergistic antioxidant, anti‐inflammatory, and proregenerative bioactivities lead to a performance for treating corneal diseases superior to that of conventional formulations. Furthermore, the innovative applications of hydrogels in postoperative protection and biosensing highlight their customizable structures and functions (**Table** **2**).

**TABLE 2:** Material–application matching guide for corneal hydrogels.

Material category	Material	Target clinical applications	Key therapeutic mechanisms	Refs.
Proteins	Collagen	Corneal stromal regeneration epithelial layer reconstruction composite artificial corneal scaffolds	Mimicry of native corneal stromal composition support of epithelial cell and keratocyte adhesion/proliferation high optical transparency	[47, 64, 66, 67]
	Gelatin	Superficial corneal injury repair drug delivery systems 3D bioprinting bioinks	Tunable mechanical properties high transparency low cost methacryloyl modification enhances stability and cell compatibility	[10, 12, 196]
	Fibrin	Temporary corneal wound sealing hemostasis postoperative adjuvant repair	Biocompatible with ECM supports cell proliferation anti‐inflammatory/antioxidant properties rapid gelation via the thrombin–fibrinogen reaction	[85, 86, 199]
	Silk fibroin	Artificial corneal substitutes corneal stromal regeneration smart wearable devices	Exceptional mechanical strength high transparency tunable degradation promotes tissue regeneration	[25, 94, 103, 202]
Polysaccharides	HA	Corneal epithelial repair sustained drug delivery sutureless defect repair	Mimics corneal ECM superior transparency refractive index matches that of the cornea rapid in situ gelation	[34, 47, 110, 115]
	Chitosan	Infectious keratitis treatment corneal wound dressings drug carriers	Inherent antimicrobial activity tissue adhesion biocompatibility transparency	[13, 118, 119, 121]
	Alginate	Irregular corneal defect filling cell carriers temporary wound sealing	Injectable ion‐crosslinkable high transparency cytocompatible tunable mechanical properties	[133, 134, 141]
	Cellulose	Corneal repair scaffolds smart sensors wearable devices	High tensile strength transparency renewability supports epithelial cells growth	[144, 145]
Animal tissues	Decellularized corneal matrix	Full‐thickness corneal defect repair artificial corneal scaffolds 3D bioprinting bioinks	Biomimetic ECM composition bioresorbable supports cell phenotype maintenance in situ gelation	[56, 79, 83, 156]
Synthetic polymers	PEG	Drug delivery systems corneal tissue engineering scaffolds Smart responsive implants	Excellent transparency tunable mechanical properties low immunogenicity Rapid gelation	[25, 32, 166, 227]
	PVA	Artificial corneal substitutes contact lenses corneal wound dressings	Superior transparency high mechanical strength resists eyelid shear forces	[22, 176, 177]
	Polyacrylic acid	Corneal sensors drug carriers 3D printing scaffolds	Tunable mechanical properties high structural stability biocompatibility photocontrollable degradation	[119, 144, 185, 186]

### 5.1 Corneal Wound Repair

As the primary protective barrier of the ocular surface, the corneal epithelium is susceptible to damage caused by physical, chemical, and biological factors. Hydrogels can form a soft and breathable protective layer that reduces secondary damage to the injured area due to eyelid friction, foreign bodies, or microorganisms. The high water content of hydrogels mimics that of the natural tear film, preventing epithelial desiccation and detachment while accelerating corneal epithelial repair. Gong et al. developed a PVA hydrogel loaded with phytic acid (PA, a natural small molecule) for treating alkali burn–induced acute epithelial injury. In vitro experiments demonstrated that this hydrogel promoted the repair of oxidative stress–induced corneal epithelial damage by inhibiting ferroptosis. In vivo experiments showed that PVA hydrogels with higher molecular weights effectively delivered PA and exhibited excellent therapeutic efficacy against corneal injury, highlighting the promising application of PVA/PA hydrogels in treating corneal epithelial defects [22]. Many hydrogels are ineffective in treating large corneal epithelial defects and suffer from poor biocompatibility or weak applicability when used as cell carriers. To address this issue, Chi et al. developed a thermosensitive hydroxypropyl chitin/carboxymethyl chitosan hydrogel exhibiting an outstanding temperature sensitivity in the range of 20°C–25°C and a transparency exceeding 80%. When applied to a rabbit model of large corneal epithelial defects (6 mm), this hydrogel notably enhanced epithelial repair and reduced inflammatory responses and scar formation, offering a novel approach for treating extensive corneal epithelial defects [240].

**FIGURE 5:**
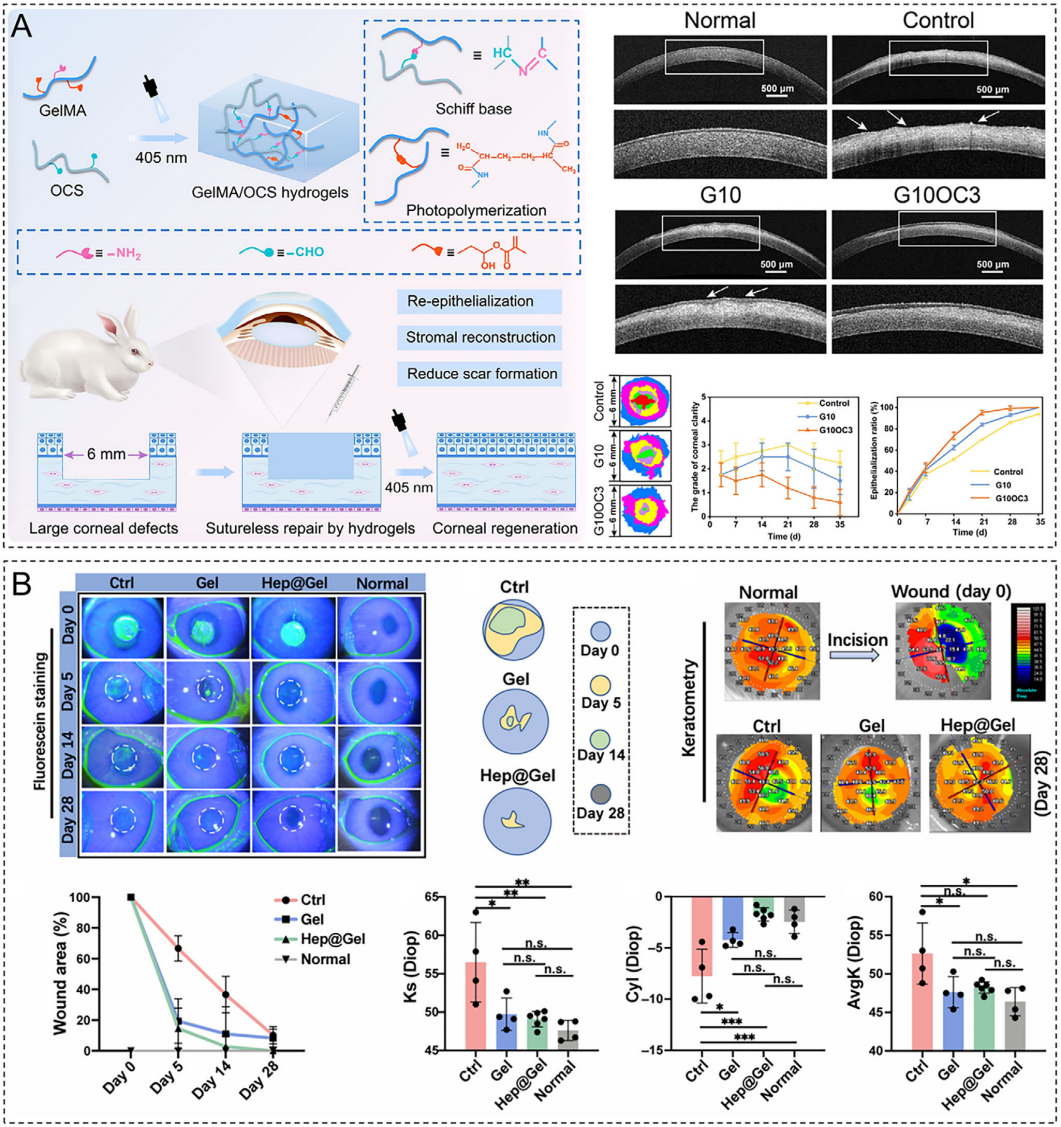
In vivo evaluation of hydrogel applications in corneal defect models. (A) Fabrication and application of photocurable gelatin methacryloyl/oxidized chondroitin sulfate hydrogels for repairing large defects. Adapted with permission [10]. Copyright 2025, Wiley‐VCH. (B) Therapeutic efficacy evaluation of bioactive hydrogels for scarless corneal repair. Adapted with permission [23]. Copyright 2024, American Association for the Advancement of Science.

During the repair of large‐area corneal defects, hydrogels not only fill the defect site but also promote the proliferation and migration of corneal epithelial cells. Li et al. developed a photocrosslinkable double‐network hydrogel based on GelMA and oxidized chondroitin sulfate (Figure 5A). This injectable hydrogel exhibited excellent transparency, a low swelling ratio, favorable mechanical properties, and superior adhesive performance, considerably promoting corneal re‐epithelialization and stromal integration/reconstruction while reducing inflammatory responses and scar formation [10]. Tissue adhesion is a critical characteristic of hydrogels used in corneal applications. For corneal wound healing, Wei et al. developed a self‐healing adhesive containing an oxidized guar gum hydrogel loaded with mesenchymal stem cell–derived exosomes. This hydrogel contained dynamic reversible Schiff base bonds between the aldehyde‐modified oxidized guar gum and carboxymethyl chitosan, thus exhibiting the shear‐thinning and self‐healing properties required for convenient needle injection. Physicochemical properties such as porosity, mechanical strength, and light transmittance could be precisely modulated by adjusting the concentration of the oxidized guar gum [195]. The above hydrogel achieved robust tissue adhesion under physiological temperatures owing to the Schiff base interactions. The encapsulated exosomes could be internalized by corneal epithelial cells to promote cell migration. Overall, this hydrogel firmly adhered to the damaged cornea, notably improved wound repair, enhanced collagen deposition, and reduced inflammation.

Under normal physiological conditions, the corneal stroma maintains transparency through well‐organized collagen fibrils. Following injury, corneal keratocytes transform into myofibroblasts, which secrete disorganized collagen fibers and the ECM, ultimately forming an opaque scar tissue. Corneal injury–induced fibrosis arises from damage to the EBM and impaired regeneration. The inhibition of corneal fibrosis and restoration of transparency depend on EBM reconstruction, during which the collagen network provides structural stability while heparan sulfate binds corneal epithelium‐derived cytokines to regulate their homeostasis. Inspired by this mechanism, Huang et al. developed a bioactive hydrogel composed of gelatin and highly anionic heparin for scarless corneal repair (Figure 5B). This hydrogel mimicked the function of the corneal EBM, blocking the activity of profibrotic factors such as IL‐1, TGF‐β, and PDGF‐BB while enabling the sustained regulation of cytokine release, and thus markedly suppressed keratocyte apoptosis and transformation into myofibroblasts [23]. In rabbit and nonhuman primate models, this hydrogel effectively restricted the influx of inflammatory and fibrotic cytokines from the epithelium into the stroma, thereby downregulating the wound‐healing cascade. Consequently, this hydrogel improved visual quality and reduced fibrosis by 73%, presenting a viable solution for scarless corneal repair. To better recapitulate the native corneal stromal microenvironment and promote scarless regeneration, Huang et al. engineered a biomimetic corneal stroma by encapsulating human amniotic epithelial stem cell‐derived keratocytes (hAESC‐SKs) within photocrosslinkable GelMA hydrogels [241]. The hAESC‐SKs not only secreted cornea‐specific proteoglycans to maintain stromal transparency but also exhibited notable immunomodulatory capabilities, reshaping the injury microenvironment by suppressing inflammatory cascades. In a rabbit corneal defect model, this bioengineered stroma facilitated rapid structural restoration and reduced scar formation by ∼75%, which highlights the potential of integrating stem cells with hydrogel scaffolds for corneal regenerative medicine.

Mitomycin‐C and 5‐fluorouracil are the primary drugs used to inhibit fibrosis. However, these drugs nonselectively suppress normal corneal cell proliferation, which raises long‐term safety concerns [242]. Wang et al. developed a mechanically stable hydrogel system based on 4XT (four tandem repeats of the immunoglobulin domain of myomesin), a recombinant protein. This composite hydrogel contained CeO_2_ nanoparticles to scavenge reactive oxygen species (ROS) and siRNA to target the expression of TGF‐β1 protein [243]. In murine corneas, this material gelled at the defect site to effectively promote wound healing while inhibiting the progression of fibrosis. By integrating the programmable and controllable properties of synthetic protein hydrogels with therapeutic strategies targeting wound mechanisms, this study provides critical insights into achieving scarless corneal wound healing. Tang et al. incorporated an anti‐inflammatory agent (dipotassium glycyrrhizinate) and an antifibrotic drug (ginsenoside Rg3) into a thermosensitive hydrogel to develop a multifunctional eye drop for the synergistic treatment of corneal alkali burns. The hydrogel network was formed through the physical and chemical crosslinking of thiolated chitosan and β‐glycerophosphate, with dipotassium glycyrrhizinate dispersed freely within the hydrogel and Rg3 embedded in liposomal form [126]. In vivo studies using murine models showed that this hydrogel effectively reduces inflammation, promotes corneal wound healing, and suppresses scarring and thus holds promise for the treatment of corneal alkali burns. Li et al. developed a thermoresponsive injectable hydrogel/nanomicelle composite as a drug delivery platform to prevent corneal scarring and reduce stromal fibrosis following lamellar keratoplasty. This in situ gelling hydrogel enables the direct delivery of celastrol (a pentacyclic triterpenoid) to the corneal stroma [52]. In vivo evaluation using a rabbit anterior lamellar keratoplasty model demonstrated the controlled release of celastrol in the corneal stroma. A single intrastromal injection of celastrol effectively alleviated fibrosis by promoting autophagy through mTORC1 signaling and suppressing the TGF‐β1/Smad2/3 pathway, which highlights the clinical potential of this hydrogel system for preventing postkeratoplasty corneal scarring and reducing stromal fibrosis. Kang et al. developed an in situ formed bioorthogonally crosslinked hydrogel for accelerated corneal regeneration. Epidermal growth factor (EGF) was conjugated to the hydrogel backbone via a photocleavable linker and could be controllably released upon exposure to mild‐intensity UV light (2—5 mW cm^−2^, 365 nm). The hydrogel gelled within minutes after application and exhibited excellent transparency and biocompatibility [110]. After UV irradiation, the hydrogel promoted corneal epithelial cell proliferation and migration in vitro. In a rat corneal wound model, UV‐triggered EGF release notably enhanced re‐epithelialization and stromal remodeling while exerting a potent antiscarring effect with minimal α‐SMA expression and strong ALDH3A1 expression. The remodeled cornea showed a completely restored thickness and lamellar architecture.

To address the challenge of simultaneous scarring and infection risks, Qie et al. developed a customized Janus hydrogel with an asymmetric architecture [244]. The bottom layer, composed of PEG and heparin, utilized electrostatic interactions to sequester proinflammatory cytokines and fibrotic factors, effectively blocking keratocyte activation. The top layer, containing spray‐coated ε‐poly‐l‐lysine, provided a robust antibacterial barrier against drug‐resistant strains. This dual‐function strategy markedly reduced fibrosis and eliminated the need for postoperative care in animal models, which highlights the potential of integrated functional hydrogels for comprehensive corneal injury management.

### 5.2 Corneal Substitutes

Penetrating keratoplasty is a widely accepted treatment for corneal perforation, a leading cause of blindness [8]. However, the scarcity of donor corneas [245] and risks such as graft failure or immune rejection remain notable challenges [246]. With their biomimetic properties and tunable mechanical characteristics matching those of the natural cornea, hydrogels are promising materials for fabricating artificial corneas. This field was pioneered by Griffith et al., who constructed the first functional human corneal equivalents using crosslinked collagen–chondroitin sulfate scaffolds, demonstrating the feasibility of promoting nerve ingrowth and epithelialization in a biosynthetic matrix [247]. Building on this pioneering work, modern ideal artificial corneas strive to integrate optical transparency, mechanical strength, permeability, processability, and recyclability. However, the existing materials exhibit limitations: poly(methyl methacrylate) suffers from a mechanical property mismatch with the corneal tissue, covalently crosslinked hydrogels are nonrecyclable and may retain residual crosslinking agents, and physically crosslinked hydrogels demonstrate poor stability.

**FIGURE 6:**
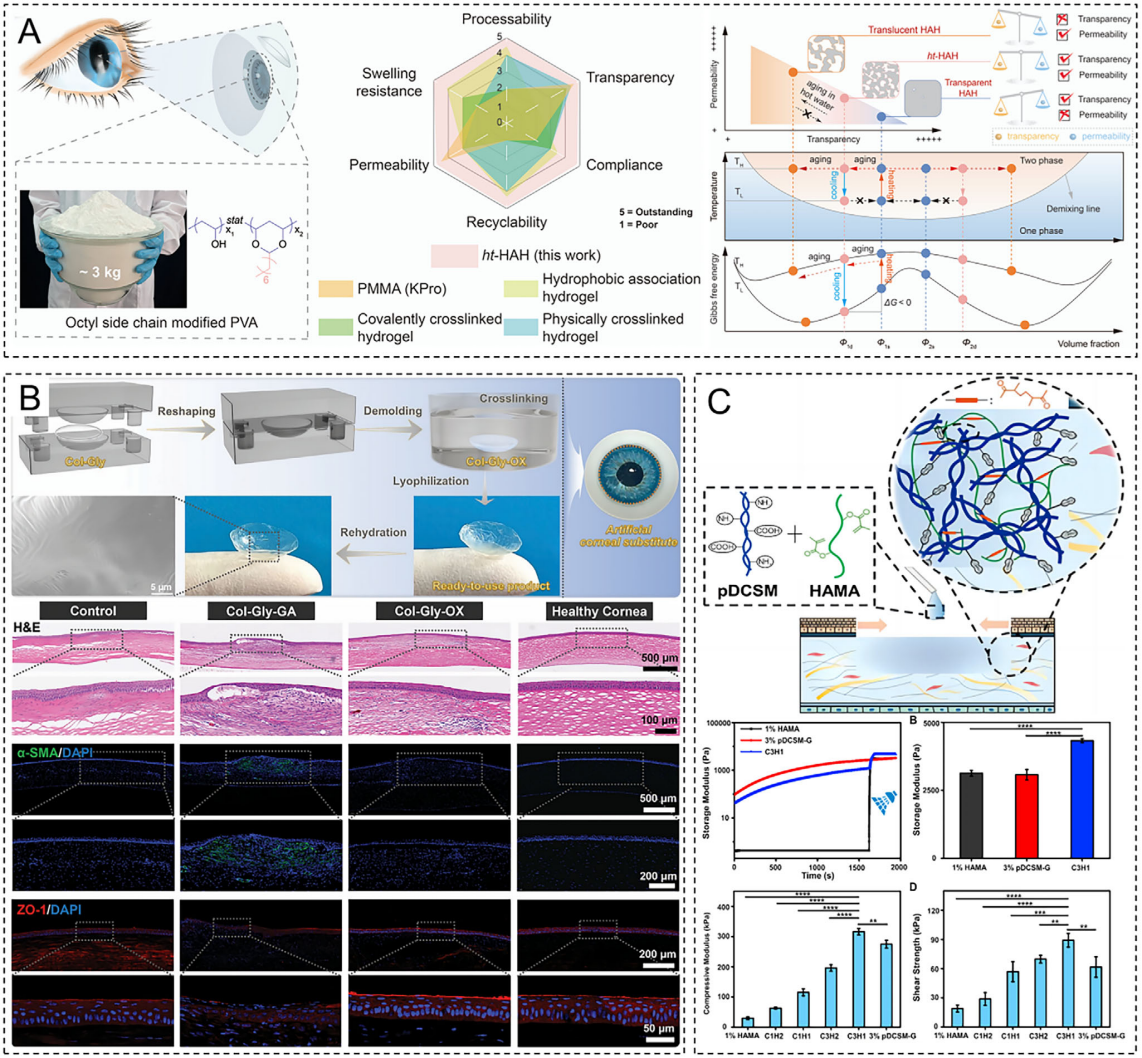
Artificial corneal substitutes for large‐area corneal defects. (A) Performance metrics of keratoprostheses fabricated via arrested phase separation. Adapted with permission [248]. Copyright 2023, Wiley‐VCH. (B) Construction of corneal substitutes and histological analysis of therapeutic efficacy. Adapted with permission [67]. Copyright 2024, Wiley‐VCH. (C) Mechanical characterization of hydrogels based on porcine decellularized corneal stroma matrix. Adapted with permission [56]. Copyright 2022, Elsevier.

Pan et al. developed an octyl side chain–modified PVA‐based material utilizing a hydrothermal treatment to induce frustrated phase separation and thereby achieve time‐dependent pore growth (Figure 6A). This approach reconciles the contradiction between permeability and transparency, enabling the optimization of multiple properties of artificial corneas. In vivo experiments confirmed the efficacy of this material in repairing corneal perforations and restoring corneal transparency, demonstrating its potential as an effective temporary substitute for corneal transplantation in emergency cases [248]. Zhao et al. designed a multifunctional hydrogel corneal patch with dual thermo‐ and photoresponsiveness. The patch comprised a dual‐crosslinked GelMA and polyethylene glycol diacrylate (PEGDA) hydrogel integrated with a natural ECM scaffold. Upon application to the ocular surface, this corneal patch spontaneously released bioadhesives at body temperature and stably adhered to the recipient cornea through photocrosslinking [249]. In vivo experiments demonstrated that the hydrogel patch‐maintained graft hydration in both full‐thickness and sutureless lamellar keratoplasty while restoring corneal structural integrity and transparency. Owing to its native matrix–like properties, versatile handling characteristics, and facile fabrication process, this hydrogel patch is a promising corneal substitute for clinical applications.

Current collagen‐based artificial corneas struggle to simultaneously meet critical requirements such as a biomimetic laminated structure, high light transmittance, sufficient thickness, and adequate permeability. Wu et al. used glycerol as a conditioning agent and oxazolidine as a crosslinking agent to construct a biomimetic corneal substitute. Glycerol disrupted and reorganized hydrogen bonds within collagen, inducing a three‐step multiscale structural evolution in the collagen hydrogel, the three steps being the reduction in molecular‐level crystallization, ordered spacing of nanoscale microfibrils, and formation of a macroscopic laminated structure. The hydrogel maintained high transmittance, oxygen permeability, biocompatibility, and mechanical properties matching those of the natural cornea even at a thickness of 1 cm, offering a green, simple, and high‐performance solution for corneal transplantation (Figure 6B) [67]. Zhao et al. developed an ion‐activated bioadhesive hydrogel for repairing large corneal defects. This hydrogel—composed of the natural corneal ECM and peptide‐modified alginate—exhibited excellent transparency, biocompatibility, tunable mechanical properties, and robust adhesion, effectively maintaining the secretory phenotype of quiescent corneal fibroblasts while preventing their transformation into myofibroblasts in vitro [141]. When applied in vivo, this hydrogel rapidly sealed 6‐mm corneal defects and, during a 6‐month follow‐up, facilitated the rapid regeneration of the corneal epithelium, stroma, and nerves and restored transparency with an efficacy comparable with that of donor corneal transplants. Hence, this material is a clinically viable scaffold for corneal surgery, particularly the repair of large defects.

Current synthetic corneal adhesives such as cyanoacrylates suffer from poor transparency and high cytotoxicity. Conversely, the use of natural hydrogel materials is mostly limited to short‐term repair with uncertain long‐term efficacy, with some natural hydrogels exhibiting poor cell adhesion. To address these limitations, Shen et al. developed a dual‐crosslinked composite hydrogel comprising porcine corneal dECM and methacrylated HA [56] (Figure 6C) using noncompetitive chemical crosslinking and photocrosslinking. This hydrogel could be directly applied to fill corneal defects of various shapes for sutureless repair. In a rabbit corneal stromal defect model, the hydrogel persisted for up to eight weeks, promoted epithelial regeneration and wound healing, and caused no notable inflammation or scar formation. Thus, the developed strategy is effective for long‐term sutureless treatment and tissue regeneration in corneal defects. Zhao et al. developed an adhesive patch mimicking the human corneal stroma for sutureless corneal transplantation. This artificial corneal substitute based on a hydrogel framework was fabricated by casting and photocuring PEGDA within a decellularized porcine corneal template followed by enzymatic digestion to obtain the hydrogel skeleton, which was subsequently combined with the human corneal ECM and GelMA [83]. The hydrogel patch replicated the ultrastructure, protein composition, and optical properties of the human cornea, demonstrating swelling and degradation resistances superior to those of conventional decellularized porcine corneas and recombinant human collagen patches. This patch released GelMA at the ocular surface temperature and achieved stable adhesion to the corneal stroma upon irradiation at 405 nm, promoting the survival and migration of corneal epithelial and stromal cells while maintaining their phenotype, reducing suture‐related complications, and preserving corneal structural stability.

### 5.3 Drug Delivery Systems

The clinical applications of traditional eye drops have several limitations, for example, the corresponding therapeutic efficacy is affected by ocular physiological barriers including tear drainage, blinking, and the corneal barrier [250]. Tear drainage and blinking shorten the retention time of eye drops and hinder drug absorption, leading to inaccurate dosing and low therapeutic efficiency. More frequent dosing may help maintain the therapeutic effects [127, 251] but reduces patient compliance and compromises the treatment outcome [252]. The frequent and high‐dose administration of eye drops can also cause ocular irritation, enzyme inhibition, bacterial resistance, and other side effects. Moreover, traditional eye drops are ineffective in adequately controlling infections or inflammation [253]. By forming a biocompatible gel layer on the corneal surface, hydrogels can overcome many of these shortcomings to achieve better drug delivery, sustained drug release/retention, and increased therapeutic efficacy while promoting tissue repair.

**FIGURE 7:**
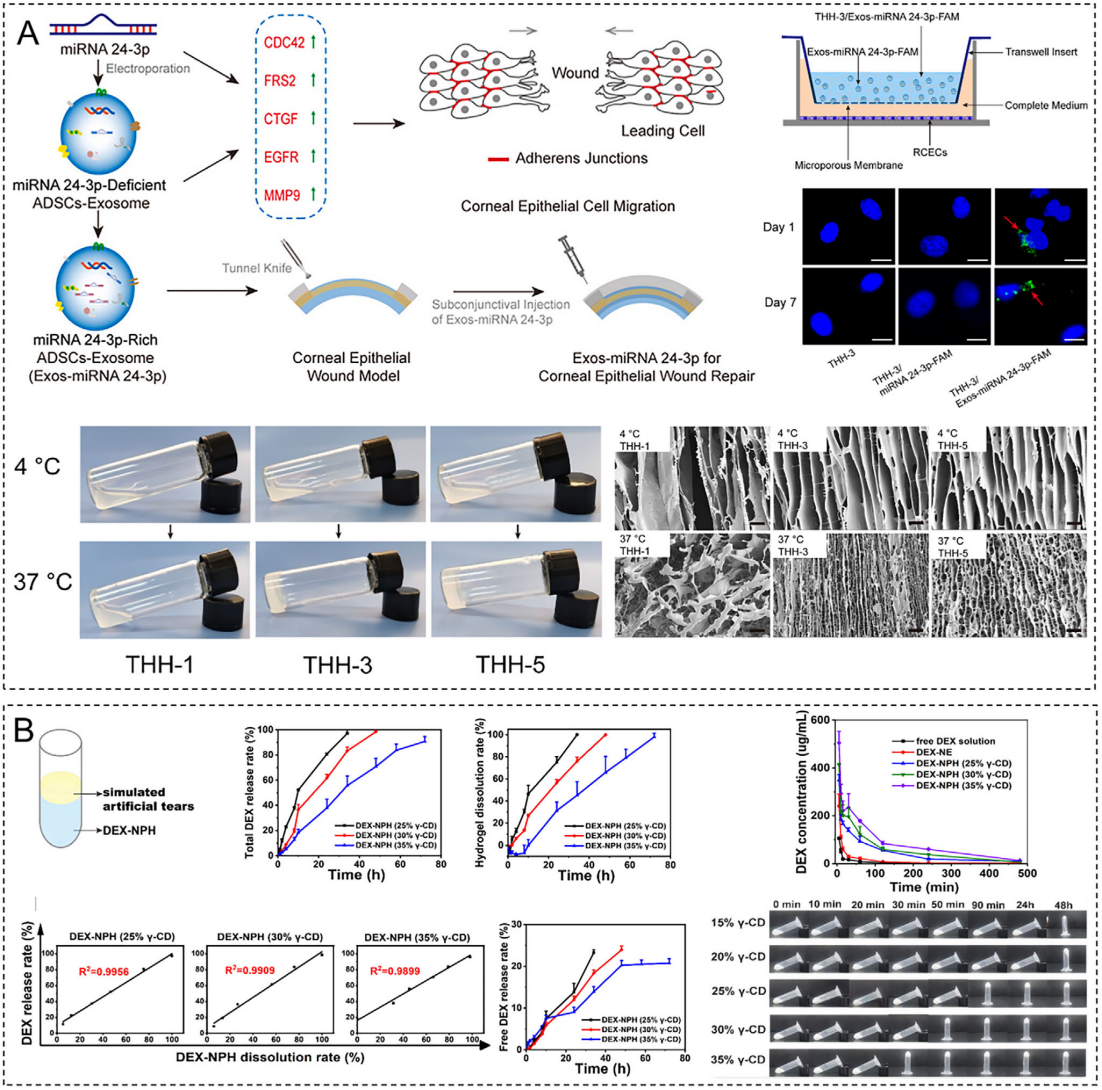
Hydrogels for corneal drug delivery. (A) Exosome‐loaded thermosensitive hydrogel for corneal epithelial healing. Adapted with permission [112]. Copyright 2022, Elsevier. (B) In vitro and in vivo drug release and degradation behaviors of dexamethasone‐loaded hydrogels with varying γ‐cyclodextrin concentrations. Adapted with permission [127]. Copyright 2025, Elsevier.

Fiorica et al. developed a hydrogel based on HA and β‐cyclodextrin to maintain corneal epithelial cell function and enable sustained dexamethasone release, revealing that the release kinetics were primarily controlled through the inclusion complexation of β‐cyclodextrin and the physical barrier effect of its 3D network [254]. The divinyl sulfone derivative of β‐cyclodextrin formed a highly stable water‐soluble inclusion complex with dexamethasone, exhibiting an apparent stability constant of 2465 m ^−1^. This high stability ensured the encapsulation of dexamethasone within the hydrophobic cavity of β‐cyclodextrin, which acted as a crosslinker while enhancing the water solubility of the drug through inclusion complexation. The drug release rate was further modulated by the dissociation kinetics of the inclusion complex. The dexamethasone inclusion complex was further embedded within the 3D network of the aminated HA hydrogel. The pore structure and swelling ratio of the hydrogel restricted free drug diffusion and, therefore, delayed outward drug migration. In vitro studies demonstrated sustained dexamethasone release for at least 5 days, and the release profile characterized by slow and continuous kinetics was consistent with the dual‐controlled release mechanism combining complex dissociation and diffusion. This system effectively addressed the challenges of hydrophobic drug loading and sustained release through β‐cyclodextrin inclusion, preventing burst release associated with direct drug loading while maintaining the biocompatibility and biodegradability of the hydrogel and thus holding promise for prolonged drug delivery in corneal wound repair. Sun et al. engineered a DEGMA‐modified hyaluronic acid hydrogel functionalized with miRNA 24‐3p‐rich exosomes [112]. This system utilized the hydrogel as a reservoir to sustain the release of bioactive exosomes, effectively promoting corneal epithelial regeneration (Figure 7A).

Although existing polymer‐based hydrogels can extend contact time, they face challenges in precise control over formulation and drug loading and may therefore cause blurred vision and affect immune responses. To address the limitations of current drug delivery systems, Pan et al. developed a supramolecular hydrogel that achieved sustained chloramphenicol release from the cornea through multiple mechanisms. Chloramphenicol was covalently conjugated with self‐assembled peptides via ester bonds to form a peptide–drug conjugate. This conjugate self‐assembled in neutral phosphate‐buffered saline to form a supramolecular hydrogel with 3D networks [251]. Chloramphenicol was embedded within this framework through covalent bonding rather than physical encapsulation; hence, its release required the hydrolysis of chemical bonds instead of simple diffusion. This hydrolysis resulted in the initial sustained release. Moreover, Ca^2+^ ions in the endogenous tear fluid formed coordination bonds with the carboxyl groups of the conjugate to afford calcium bridges modulating the elasticity and porosity of the hydrogel. Compared with traditional polymeric hydrogels, this supramolecular hydrogel leveraged covalent conjugation, ionic modulation, and enzymatic responsiveness, offering a novel strategy for sustained ocular drug delivery.

Current steroid eye drops face similar limitations. Their frequent administration increases the risk of side effects (e.g., steroid‐induced glaucoma) and reduces patient compliance. Although nanoemulsions can improve the solubility and permeability of lipophilic drugs, their low viscosity results in a short ocular residence time. Conversely, hydrogels are capable of extending the retention time and resisting clearance from blinking but often underperform in the loading of hydrophobic drugs. To address the shortcomings of existing steroid drug delivery platforms, Fang et al. developed a nanoemulsion‐based pseudopolyrotaxane hydrogel loaded with dexamethasone for treating corneal inflammation (Figure 7B). This hydrogel was formed through host–guest interactions between γ‐cyclodextrin and Tween 80 nanoemulsions, along with hydrogen bonds crosslinking γ‐cyclodextrin molecules. In in vitro experiments, the drug release rate was highly correlated with the hydrogel erosion rate, which was modulated by the concentration of γ‐cyclodextrin. Higher γ‐cyclodextrin concentrations resulted in a denser hydrogel network, slower erosion, and more sustained drug release [127]. Release medium analysis revealed that >75% of the released drug remained encapsulated within the nanoemulsions rather than existing in the free form. Nanoemulsions not only enhanced drug solubility but also prolonged drug efficacy through their inherent sustained‐release properties. Furthermore, the hydrogel acted as a nanoemulsion carrier and physically restricted nanoemulsion diffusion, whereas gradual hydrogel erosion enabled the controlled release of the nanoemulsion to achieve dual sustained release. Thus, this strategy holds promise for enhancing the bioavailability of drugs at the cornea and treating infectious keratitis.

In summary, hydrogels can prolong the residence time of drugs on the ocular surface, enabling drug accumulation and sustained release to maintain therapeutic concentrations and thus reducing the need for frequent drug administration. Moreover, the bioavailability of drugs in hydrogels can be enhanced by optimizing their rheological properties, crosslinker concentration, and bioadhesive properties.

### 5.4 Smart Responsive Hydrogels

Responsive hydrogels enable targeted and controlled drug release through physical or chemical stimuli, thereby enhancing therapeutic precision, optimizing treatment efficacy, and minimizing side effects. These hydrogels can also simulate the microenvironment of the corneal tissue, supporting cell growth/regeneration and effectively repairing damaged corneas while maintaining their transparency and structural integrity [41, 255]. Furthermore, their dynamic response mechanisms meet the diverse requirements of corneal wound healing, offering highly customizable therapeutic approaches in tissue engineering and regenerative medicine [13].

**FIGURE 8:**
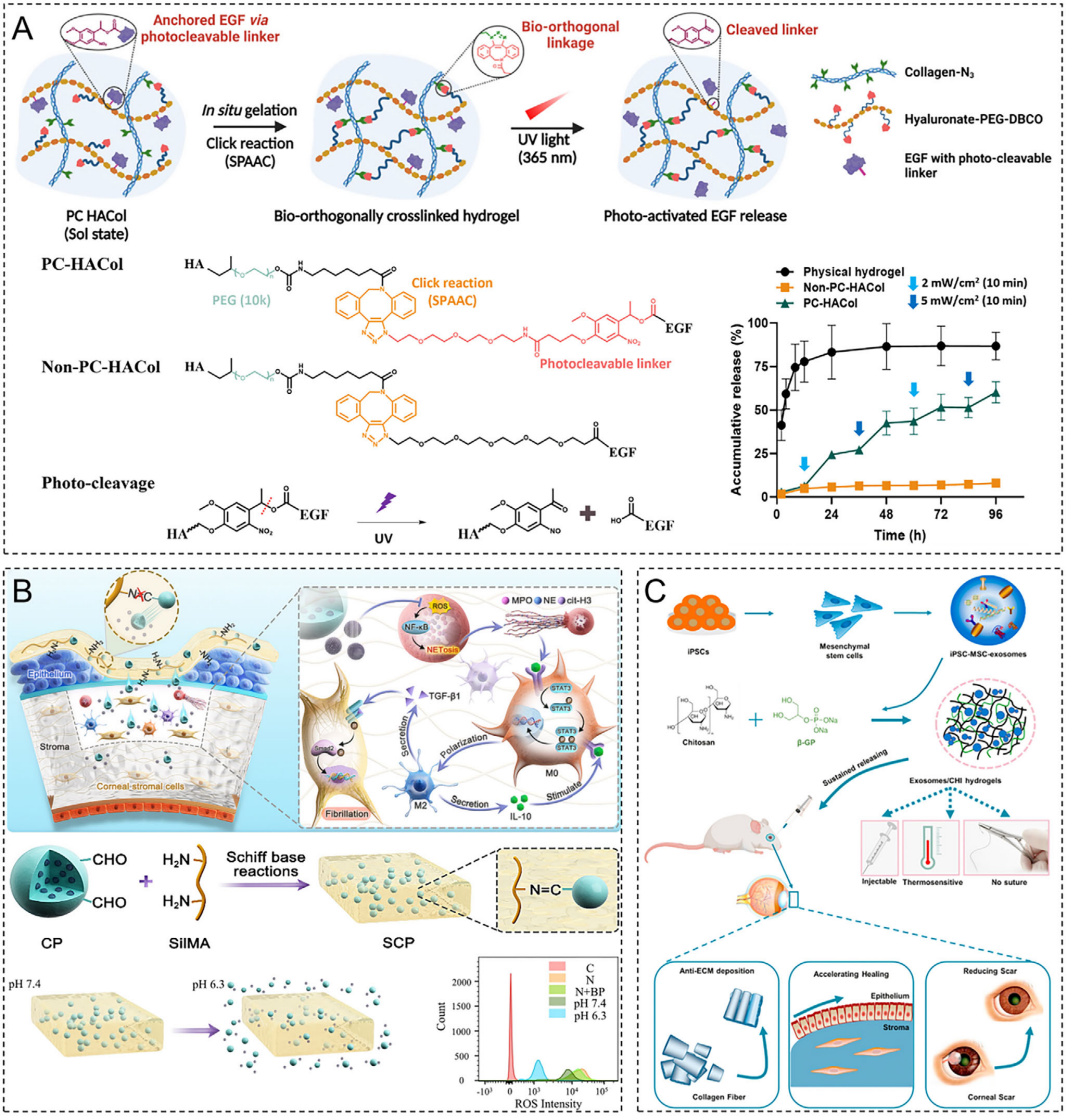
Stimuli‐responsive hydrogels for corneal applications. (A) Light‐responsive hydrogel for epidermal growth factor release in corneal defect regeneration. Adapted with permission [110]. Copyright 2024, Elsevier. (B) pH‐responsive hydrogel inhibiting the innate immune cascade fibrosis after corneal chemical injury. Adapted with permission [257]. Copyright 2023, Elsevier. (C) Action mechanism of an exosome‐loaded thermoresponsive hydrogel. Adapted with permission [125]. Copyright 2021, Elsevier.

In situ–forming thermosensitive hydrogels can undergo rapid gelation triggered by body temperature, achieving precise adherence to corneal wounds through dropwise administration. Owing to their capability of drug loading for intelligent controlled release, these hydrogels respond to inflammation or temperature variations to deliver therapeutic agents, eliminating the need for frequent eye drop instillation and offering an efficient, precise, and patient‐friendly innovative solution for ophthalmic treatment. Yan et al. developed a temperature‐responsive smart hydrogel based on an amphiphilic thermosensitive polymer—Pluronic F‐127 (PEG‐polypropylene glycol‐PEG triblock copolymer)—and a biomacromolecule (progranulin) for treating corneal alkali burns [256]. In aqueous solution, Pluronic F‐127 exhibits a pronounced sol–gel transition in response to temperature changes. Up to 25°C, the hydrophilic PEG segments are dominant, enabling the molecules to disperse as free chains in a liquid state and facilitating progranulin loading and ocular surface administration. When the temperature reaches ∼34.5°C, the increased hydrophobicity of the polypropylene glycol segments drives intermolecular aggregation into micellar networks, which rapidly transforms the solution into a gel state and affords an adhesive coating on the corneal surface. Capitalizing on the thermosensitive sol–gel transition of Pluronic F‐127, the hydrogel functions as a dynamic system characterized by low‐temperature injectability, rapid in situ gelation at physiological temperatures, and subsequent sustained drug release. The result is a microenvironment at corneal injury sites that integrates physical protection and biological regulation, providing an efficient and low‐toxicity therapeutic strategy for severe diseases at the ocular surface such as corneal alkali burns. Tang et al. developed a thermosensitive chitosan and β‐glycerophosphate–based hydrogel loaded with mesenchymal stem cell exosomes (Figure 8C). Chitosan exists in a molecularly dispersed form in solution at low temperatures. With increasing temperature, a 3D network structure between chitosan and β‐glycerophosphate is formed through noncovalent interactions such as hydrogen bonds and hydrophobic interactions, leading to gelation [125]. The hydrogel achieves reversible sol‐gel transitions through temperature‐driven intermolecular interactions, maintaining fluidity at low temperatures for easy administration and solidifying into a gel at physiological temperatures to function as a drug carrier. This thermosensitive responsiveness meets the clinical demands for corneal injury repair.

Following ocular chemical injury, chemical stimuli induce the accumulation of ROS in neutrophils, activating the NF‐κB pathway and accelerating the formation of neutrophil extracellular traps (NETs). As a critical mediator, NETs promote M2 macrophage polarization via the IL‐10/STAT3 pathway. These M2 macrophages activate the TGF‐β1/Smad2 pathway in corneal stromal cells, driving their transformation into fibroblasts and ultimately leading to corneal stromal fibrosis, which impedes corneal repair. Based on this injury mechanism, Zhang et al. developed a functional pH‐responsive hydrogel by integrating sericin with oxidized chitosan nanoparticles loaded with black phosphorus quantum dots (BPQDs; Figure 8B) [257]. In this hydrogel, the aldehyde groups of oxidized chitosan form dynamic covalent bonds (Schiff base bonds, ─C═N─) with the amino groups of methacrylated sericin. The stability of these bonds depends on environmental pH. After chemical injury, localized acute inflammation triggers an innate immune response, creating an acidic microenvironment. This acidic condition causes the Schiff base bonds to break, leading to hydrogel disintegration and the selective release of BPQDs. In contrast, under neutral conditions, the Schiff base bonds remain stable, suppressing the release of BPQDs. This pH‐dependent release mechanism ensures the precise delivery of BPQDs to the injured area while minimizing off‐target effects on healthy tissues. The released BPQDs scavenge ROS, inhibit NET formation, and disrupt the innate immune‐driven fibrotic cascade, ultimately mitigating pathological corneal fibrosis and promoting ocular surface reconstruction.

Owing to their photocontrollable properties, light‐responsive hydrogels enable noncontact and spatiotemporally precise drug release or mechanical modulation, thus reducing invasive interventions. Phototriggered gelation further enables minimally invasive in situ hydrogel formation, facilitating personalized adaptation to complex corneal defects. These advancements offer intelligent solutions for corneal disease treatment, wound repair, and the development of transplantation materials. Kang et al. developed a photoresponsive hydrogel based on HA derivatives and azide‐functionalized collagen for corneal injury regeneration (Figure 8A) [110]. In this system, EGF is covalently conjugated to the HA‐PEG‐dibenzocyclooctyne backbone via an *o*‐nitrobenzyl‐derived photocleavable linker sensitive to mild‐intensity UV light (365 nm, 2–5 mW cm^−2^). Upon exposure to UV irradiation under these conditions, the linker is cleaved to enable the controlled release of EGF from the hydrogel network. The EGF release quantity and rate could be precisely regulated by modulating the duration, intensity, and frequency of UV exposure. In vitro experiments demonstrated that prolonged exposure and higher irradiation intensity resulted in increased EGF release. In vivo experiments further revealed that higher irradiation frequencies notably accelerated corneal epithelial regeneration. Notably, the employed UV intensity was lower than that of natural sunlight and comparable with that used in Food and Drug Administration (FDA)‐approved protocols for crosslinking corneal collagen, thereby reducing the risk of UV‐induced damage to corneal tissues. Additionally, this hydrogel exhibited rapid in situ gelation at the corneal defect sites, seamlessly adapting to the wound morphology. These features enabled the intelligent regulation of the corneal repair process to achieve precise responsiveness and excellent biocompatibility. Yazdanpanah et al. prepared a photoresponsive hydrogel by reacting a thermoresponsive porcine corneal dECM with methacrylic anhydride and thereby grafting methacrylate groups onto the free amine groups of collagens, sulfated glycosaminoglycans, and proteoglycans. This modification endowed the hydrogel molecular chains with the capacity for photocrosslinking without notably altering the original composition of collagen and sulfated glycosaminoglycans, thus retaining compositional characteristics similar to those of native corneal stroma [156]. Subsequently, an FDA‐approved photoinitiator system comprising eosin Y, triethanolamine, and *N*‐vinylcaprolactam was incorporated. Exposure to green light (520 nm, the peak absorption wavelength of eosin Y) activated the photoinitiator, triggering covalent crosslinking reactions between methacrylate groups and a liquid‐to‐solid phase transition of the hydrogel. This photoresponsive process exhibited excellent controllability, with the degree of crosslinking depending on the irradiation time and intensity. Such tunability allowed the hydrogel to precisely adapt to the repair requirements of corneal defects during surgery and avoid issues such as excessive diffusion or insufficient crosslinking.

Despite these advantages, the use of stimulus‐responsive hydrogels in the treatment of corneal diseases faces numerous challenges. In real‐world clinical scenarios, the ocular surface environment is highly complex and dynamic, which may lead to nonspecific or delayed responses and thereby reduce the controllability of drug release or hydrogel structural changes. Further improvements are needed to enhance response precision under intricate physiological conditions.

## 6 Integration of Cutting‐Edge Technologies

### 6.1 3D Bioprinting Technology

As an emerging additive manufacturing technology, 3D bioprinting encompasses techniques such as fused deposition modeling, direct ink writing, selective laser melting, digital light processing (DLP), and stereolithography and is designed to fabricate 3D tissues or organs with intricate geometries and biological functionalities [258, 259]. These constructs are composed of bioinks, which are combinations of biocompatible materials and living cells and are precisely deposited in predefined 3D configurations to form microbiologically active materials or tissue scaffolds constructed from human cells [260, 261, 262]. Hydrogels have emerged as predominant bioink components owing to their high biocompatibility, ECM‐mimicking properties, and tunable physicochemical characteristics [263, 264, 265]. Common hydrogel‐based bioinks contain gelatin, collagen, HA, alginate, and PEG [266].

The clinical applicability of existing hydrogel scaffolds is limited by curvature mismatch, poor mechanical properties, and insufficient ROS scavenging capacity. To address these challenges, Li et al. developed a three‐dimensionally printed nanocomposite hydrogel scaffold for corneal repair [267]. The employed hydrogel comprised GelMA, PEGDA, laponite, and dopamine. GelMA enhanced cell adhesion, proliferation, and optical transparency, and PEGDA improved mechanical strength and reduced swelling. Laponite enhanced rheological properties by increasing stiffness and toughness, and dopamine provided ROS scavenging capability and acted in synergy with the nanoclay to improve mechanical performance. After mixing the components, the scaffold was fabricated via extrusion‐based 3D printing and crosslinked by exposure to 365 nm UV light for 30 s. This scaffold exhibited well‐matched curvature, superior mechanical and ROS scavenging properties, and excellent biocompatibility, mitigating oxidative stress damage and promoting cell survival and proliferation and thereby accelerating epithelial regeneration and stromal repair while reducing the risk of scar formation. Highly efficient corneal restoration was achieved by precisely matching curvature and thickness for each individual corneal defect.

**FIGURE 9:**
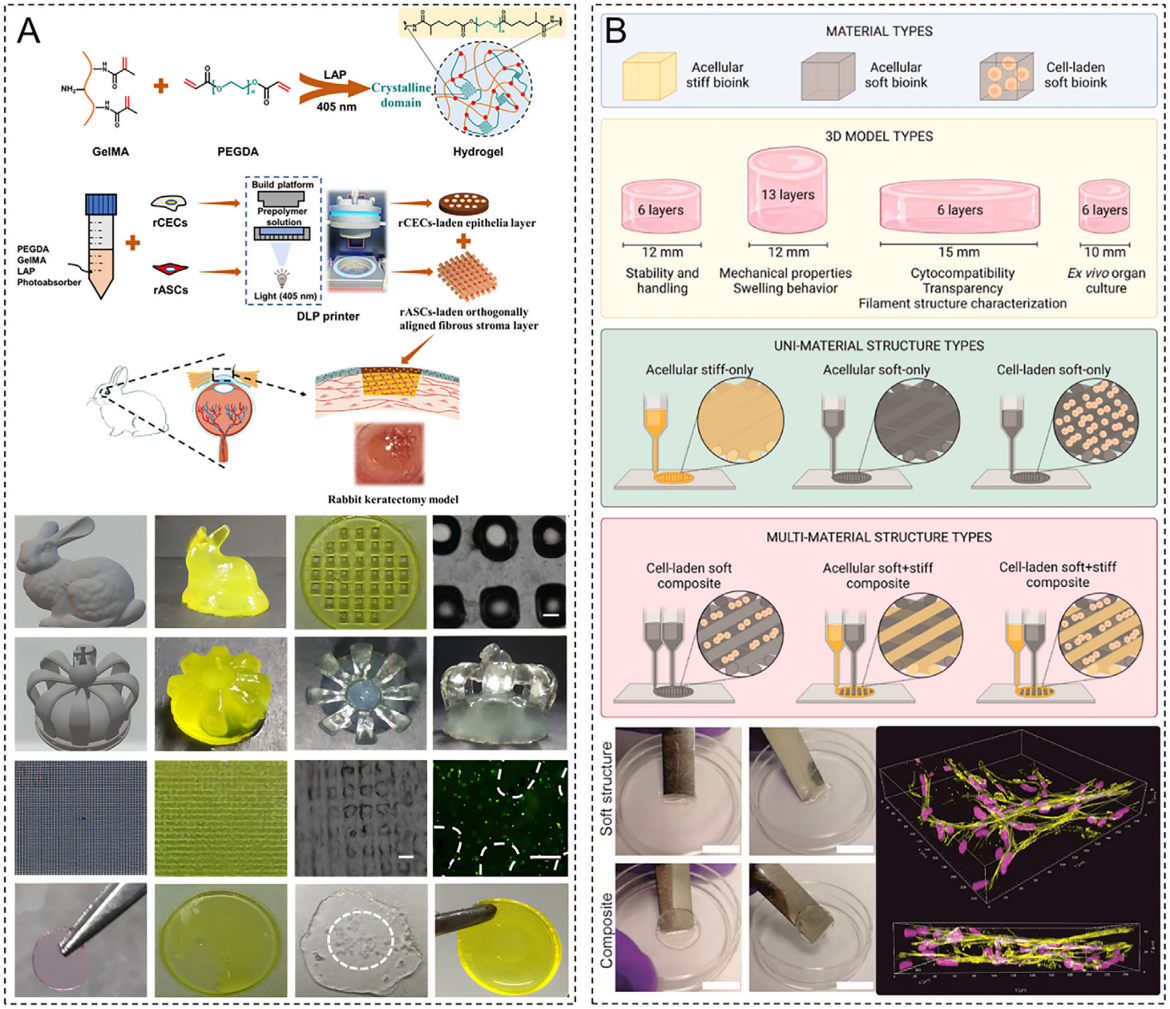
Three‐dimensionally printed hydrogel for corneal applications. (A) Schematic of three‐dimensionally printed biomimetic epithelium/stroma bilayer hydrogel and printability evaluation. Adapted with permission [268]. Copyright 2022, Elsevier. (B) Multimaterial three‐dimensional bioprinting strategy and cytocompatibility within the bioprinted composite structure. Adapted with permission [269]. Copyright 2023, Elsevier.

Traditional hydrogel corneal scaffolds face challenges such as complex fabrication processes, the inability to mimic multilayer structures, and poor mechanical and optical properties. Unlike extrusion‐based 3D printing, which suffers from low resolution and high cell damage, DLP offers the advantages of superior resolution, cell viability, and speed. To address the structural complexity of corneal regeneration and the issue of corneal cell–fibroblast transformation, He et al. developed a biomimetic epithelial/stromal bilayer implant using 3D printing (Figure 9A). A two‐component bioink was formulated by blending gelatin methacrylate with long‐chain PEGD, and a hydrogel‐based corneal implant was fabricated using DLP [268]. DLP printing relies on the layer‐by‐layer photopolymerization of liquid bioinks, achieving high precision and resolution (∼1 µm). This implant enabled the precise construction of orthogonally aligned fibrous structures in the corneal stromal layer, mimicking the native corneal microstructure. As cell damage during printing was avoided owing to the minimal shear stress, the postprinting cell viability exceeded 90%. Extrusion‐based printing requires small nozzles (<100 µm) that result in low printing speeds, easily damage cells, and are poorly suited for forming complex curved surfaces, whereas DLP operates at high speeds and produces hydrogels with curved and smooth surfaces, high light transmittance, appropriate swelling properties, nutrient permeability, and suitable degradation rates. The printed bilayer dome‐shaped corneal scaffold featured an epithelial layer loaded with rabbit corneal epithelial cells and a stromal layer composed of orthogonally aligned fibers loaded with rabbit adipose tissue‐derived mesenchymal stem cells. In a rabbit corneal transplantation model, this scaffold achieved effective corneal defect sealing, re‐epithelialization, and stromal regeneration. The synergistic effects of its microstructure and precise cell positioning resulted in an optimized microenvironment for corneal regeneration and excellent biocompatibility.

Existing 3D printing technologies primarily focus on the shape, mechanical properties, or optical performance of the cornea but often fail to replicate heterogeneous microstructures, which leads to suboptimal tissue formation. Single‐material bioprinting struggles to mimic the heterogeneity of native tissues. Puistola et al. developed a multimaterial 3D bioprinting strategy that simulates the interlayer orthogonal arrangement of collagen fibers in the corneal stroma and cell alignment along these fibers and used it to fabricate an artificial corneal stroma with a heterogeneous design (Figure 9B) [269]. This strategy employed human adipose tissue‐derived stem cells (hADSCs) and HA‐based bioinks of varying stiffness. By alternately printing cell‐laden soft and cell‐free stiff HA bioinks in orthogonally layered fibers, the researchers replicated the heterogeneous microstructure of the native corneal stroma. The soft bioink promoted hADSC proliferation and tissue formation, whereas the stiff bioink provided mechanical support and guided cell alignment. The mechanical properties of the construct notably improved with cell growth and demonstrated excellent integration with the host tissue in an ex vivo porcine corneal model. This innovative solution advances corneal tissue engineering by addressing the limitations of conventional bioprinting methods.

Overall, 3D printing can achieve fine control unattainable through conventional methods, enabling the fabrication of corneal scaffolds with highly precise dimensions, patterns, and complex architectures. This technique can produce hydrogel structures with minimal deformation and exceptional dimensional accuracy, ensuring optimal integration and matching with native corneal tissues [270]. As matrix materials for 3D printing, hydrogels provide a highly biocompatible microenvironment that supports corneal cell adhesion, proliferation, and functional expression [271]. Dynamically crosslinked hydrogels further facilitate four‐dimensional (4D) printing, enabling shape adaptation in response to hydration changes and thereby accommodating the dynamic corneal repair process [272, 273]. However, the high‐water content of hydrogels may compromise their mechanical strength, with posthydration softening reducing corneal structural stability [274, 275]. The mechanical properties of three‐dimensionally printed scaffolds are often enhanced through secondary crosslinking, which, however, may adversely affect biocompatibility [137]. The safety and selectivity of photoinitiators are important parameters for photopolymerization‐based 3D bioprinting. The insufficient photosensitivity of certain materials can compromise printing precision and biosafety [276, 277]. Traditional extrusion‐based printing faces limitations in the processing of soft materials, for example, structural deformation, high interlayer adhesion that restricts printing speed, and poor stability of large‐scale constructs [278, 279]. The intricate microstructure of the cornea demands ultrahigh precision; however, current technologies struggle to balance resolution with material rheology [280].

### 6.2 Corneal Wearable Devices

With the rapid advancement of smart wearable devices, hydrogels have emerged as a research hotspot owing to their unique properties, demonstrating broad prospects of application in flexible sensors, energy supply, and human–machine interactions. Hydrogels incorporating conductive polymers, ionic liquids, or nanomaterials can simultaneously achieve high flexibility, biocompatibility, and good electrical conductivity and are ideal for fabricating wearable flexible sensors. For example, devices capable of simultaneous piezoresistive and piezoelectric sensing allow the precise monitoring of human motion and physiological signals. Such sensors can track body movements, physiological parameters, and environmental stimuli in real time, thereby facilitating personalized health management [281, 282, 283]. Owing to their prolonged interactions with the cornea, hydrogel‐based contact lenses serve as potential drug delivery platforms by extending drug retention on the ocular surface, enhancing bioavailability, and maintaining sustained therapeutic drug concentrations [284, 285]. Furthermore, smart hydrogel lenses enable personalized treatment by monitoring metabolites (e.g., glucose and inflammatory markers) in real time, thereby advancing integrated diagnosis and therapy for conditions such as glaucoma and corneal injuries [45, 286].

**FIGURE 10:**
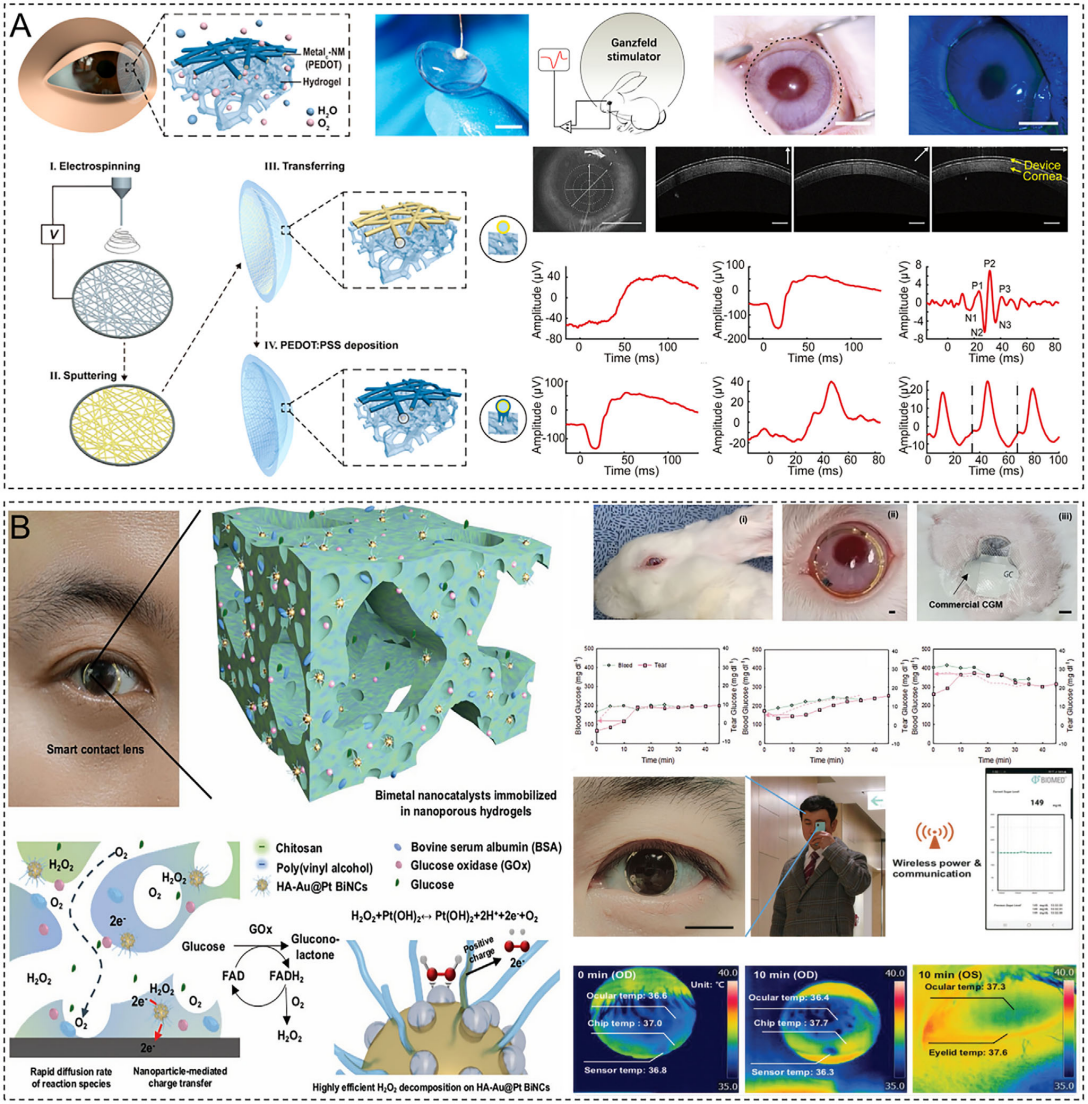
Corneal wearable smart devices. (A) Eye‐interfacing device based on hydrogel contact lenses for full‐field electroretinogram recording. Adapted with permission [287]. Copyright 2019, American Chemical Society. (B) Hydrogel‐based smart contact lens for long‐term continuous glucose monitoring. Adapted with permission [288]. Copyright 2022, Wiley‐VCH.

Existing contact lens‐based electronic devices often consist of substrates, electronic components, and adhesive layers, and their long‐term daily wear is hindered by poor breathability. For example, graphene‐based devices exhibit inadequate oxygen permeability and wettability, leading to deposits and discomfort. Plastic substrates are flexible but also insufficiently breathable. Wei et al. designed an ocular interface of a hydrogel contact lens with high breathability, wettability, hydration levels, optical transparency, mechanical compliance, and durability (Figure 10A) [287]. This device utilized commercially available hydrogel contact lenses as the substrate, metal‐coated nanofiber meshes as conductors, and in situ electrochemically deposited poly(3,4‐ethylenedioxythiophene)/poly(styrene sulfonate) as an adhesive material. In a rabbit model, the continuous wearing of this device for 12 h resulted in a corneal fluorescein staining level comparable with that observed for pure‐hydrogel lenses and caused no notable corneal damage or irritation, which confirmed high device safety. The functional efficacy of this device was validated by recording full‐field electroretinograms in rabbit eyes. This integrated strategy based on nanofiber mesh electronics provides a versatile platform for hydrogel‐based electronic devices, enhancing the biocompatibility of wearable or implantable sensors. By overcoming the breathability limitations of existing devices, it achieves high safety, daily wearability, and multifunctional ocular interfacing, thereby offering a universal platform for ocular diagnostics, augmented reality, and related fields.

Continuous glucose monitoring is critical for diabetes management. To overcome the limitations of conventional monitoring methods, Kim et al. developed a smart contact lens primarily comprising a nanoporous hydrogel and an HA‐Au@Pt bimetallic nanocatalyst (Figure 10B) [288]. After glucose oxidase reacts with glucose to generate H_2_O_2_, the HA‐Au@Pt nanocatalyst rapidly decomposes H_2_O_2_ into O_2_ and electrons, enhancing charge transfer while mitigating the H_2_O_2_‐induced inhibition of glucose oxidase. The nanoporous hydrogel accelerates glucose diffusion in tears, addressing the issues of low tear volume and slow diffusion and ensuring rapid analyte delivery to the sensor. These synergistic mechanisms achieve low hysteresis, allowing tear glucose levels to accurately reflect blood glucose trends. Finally, the detection results are transmitted wirelessly and stably using data synchronized to smartphones. In addition to functionality, this contact lens exhibited excellent biocompatibility and high oxygen permeability. In a diabetic rabbit model, tear glucose levels measured using this lens well correlated with blood glucose levels, with 94.9% of the data falling within clinically acceptable ranges. These results lay a foundation for clinical application.

Color vision deficiency (CVD) is a congenital ocular disorder most commonly manifested as red–green color blindness. Current treatments primarily rely on tinted lenses to enhance color perception; however, dyed lenses pose the risks of dye leakage and toxicity. Salih et al. designed an Au nanocomposite contact lens composed of PHEMA and Au nanoparticles (AuNPs) for managing red–green CVD [289]. AuNPs with finely tuned sizes leverage surface plasmon resonance properties to selectively filter light in the 540–580 nm range, which is difficult to distinguish for people with red–green CVD. After integration into a hydrogel matrix, the composite lens exhibited a transmission spectrum similar to that of commercial color‐correcting devices, effectively blocking light in the red–green crossover region while maintaining normal transmission at other wavelengths to improve color discrimination. Although water retention minimally decreased at higher AuNP concentrations, it remained superior to that of some commercial lenses. This lens demonstrates good biocompatibility, holding promise for CVD management and spectral filtering applications.

Although most smart corneal devices remain in the research phase, considerable progress has been made in clinical translation and commercialization. SENSIMED Triggerfish (Sensimed AG), a CE‐marked and FDA‐approved smart contact lens, represents a pioneering clinical application in glaucoma management [290]. This contact lens integrates a strain gauge to continuously monitor ocular dimensional changes correlated with intraocular pressure fluctuations over 24 h, providing data that traditional tonometry cannot capture [291]. In the realm of therapeutic delivery, ACUVUE Theravision (Johnson & Johnson Vision Care, Inc.) has emerged as the first FDA‐approved drug‐eluting contact lens [292]. This etafilcon A–based hydrogel lens contains ketotifen, an antihistamine, effectively managing allergic conjunctivitis itch for up to 12 h while correcting refractive errors [293]. These commercial successes validate the feasibility of using hydrogel‐based wearable devices in clinical settings, although the realization of next‐generation multifunctional integrated systems faces challenges regarding long‐term power supply and on‐chip data processing.

## 7 Summary and Outlook

This review systematically summarizes the multifaceted progress in the corneal applications of hydrogels, covering their core properties, material classifications, functional applications, and integration with cutting‐edge technologies. Owing to their 3D network structure, high water content, and excellent biocompatibility, hydrogels have emerged as pivotal biomaterials addressing unmet clinical needs in corneal repair, artificial substitutes, drug delivery, and smart devices. From the perspective of material design, natural hydrogels excel in biocompatibility and biomimicry, while synthetic hydrogels offer the benefits of tunable mechanical properties and batch consistency, with dECM hydrogels replicating native corneal microenvironments. Functionally, hydrogels have demonstrated remarkable efficacy in promoting scarless wound healing, serving as sutureless corneal substitutes, enabling sustained drug release to overcome the limitations of traditional eye drops, and acting as stimuli‐responsive platforms for personalized therapy. The integration of 3D bioprinting and wearable technologies further expands the application scope of hydrogels, allowing the fabrication of biomimetic corneal scaffolds with precise microstructures enabling the real‐time monitoring of ocular physiology. However, bridging the gap between benchtop innovation and clinical translation necessitates overcoming several critical limitations.

Fundamentally, the design of corneal hydrogels faces a material property trilemma involving mechanical strength, optical transparency, and biocompatibility. The native cornea achieves its robustness and clarity through a unique anisotropic orthogonal collagen arrangement, a structure that is difficult to fully recapitulate in isotropic hydrogel networks. Increasing crosslinking density to match the tensile strength of the cornea often compromises water content and nutrient permeability, leading to cornea hypoxia or implant opacity. Future research must focus on advanced fabrication techniques, such as multimaterial 3D bioprinting, to engineer anisotropic microstructures that simultaneously satisfy load‐bearing and optical requirements without sacrificing bioactivity. Additionally, achieving robust long‐term adhesion in the wet and dynamic ocular surface environment remains a persistent challenge. Although recent bioadhesives have shown promise, developing sutureless interfaces that can withstand eyelid shear forces and intraocular pressure fluctuations while degrading synchronously with tissue regeneration cycles requires further innovation in dynamic covalent chemistry and topological adhesion strategies.

Regarding clinical translation, the standardization and sterilization of hydrogel products present substantial technical difficulties. Natural polymer‐based hydrogels are biocompatible but suffer from batch‐to‐batch variability and sensitivity to standard sterilization methods, which can denature bioactive proteins or degrade polysaccharide chains. Developing nondestructive sterilization protocols and establishing rigorous quality control standards for immunogenicity and degradation byproducts are prerequisites for regulatory approval. Furthermore, the transition from single‐function scaffolds to multifunctional platforms is essential. Effective treatment of severe pathologies, such as infectious keratitis or chemical burns, demands hydrogels that not only provide structural support but also modulate the immune microenvironment, suppress neovascularization, and combat drug‐resistant bacteria.

Looking forward, the development of corneal hydrogels will focus on intelligent functionalization, structural biomimicry, and clinical translatability. Intelligent hydrogels responsive to multiple stimuli will enable spatiotemporally controlled drug release and dynamic adaptation to the healing microenvironment, addressing the heterogeneity of corneal injuries. Advances in structural biomimicry, driven by 3D/4D bioprinting and microfabrication technologies, will facilitate precise cell alignment and the replication of the hierarchical lamellar structure of the cornea to enhance tissue integration and functional restoration. Material limitations will be overcome by prioritizing hybrid hydrogel systems combining natural polymers, synthetic materials, and bioactive components, and leveraging the strengths of each component to achieve optimal biocompatibility, mechanical performance, and bioactivity. For clinical translation, establishing standardized evaluation criteria for hydrogel biocompatibility, optical properties, and long‐term safety is essential, along with the development of scalable manufacturing and sterilization protocols. Furthermore, the integration of artificial intelligence in hydrogel design and wearable sensing technologies will enable personalized treatment strategies, from customized corneal substitutes to the real‐time monitoring of therapeutic responses. Ultimately, the goal is to advance hydrogel‐based therapies from passive tissue replacement to active intelligent reconstruction and thus provide a definitive solution to global corneal donor shortages and restore full visual and sensory function in patients with corneal diseases.

## Author Contributions

Xinwei Wang: visualization, validation, and writing the original draft. Wen Zhou: methodology, supervision, and investigation. Haotian Xue: writing the review and editing, conceptualization, and data curation. Wenjun Xue: investigation, data curation, and conceptualization. Guangping Gao: investigation and data curation. Bing Zhu: data curation and conceptualization. Hongyuan Song: funding acquisition, data curation, and conceptualization. Qingqiang Xu: writing the review and editing, funding acquisition, and supervision. Jing Zhao: writing the review and editing, funding acquisition, formal analysis, and resources. Wei Shen: writing the review and editing, funding acquisition, formal analysis, resources, and conceptualization.

## Funding

This work was supported by the National Natural Science Foundation of China (82271106, 82273672, 82471116, 82271119, 82571241), Shanghai Rising‐Star Program (23QA1401000), and the Shanghai Oriental Talents Project Youth Project (ONKJ2024055).

## Conflicts of Interest

The authors declare no conflicts of interest.

## Data Availability

No data were used for the research described in the article.
